# Personalization and Fortification of In‐Space Manufactured Emulsified Beverages for Long‐Term Space Travel

**DOI:** 10.1111/1541-4337.70598

**Published:** 2026-08-28

**Authors:** Svenja Schmidt, Ian Fisk, Ni Yang, Maria Saarela, Volker Hessel

**Affiliations:** ^1^ School of Chemical Engineering Adelaide University Adelaide South Australia Australia; ^2^ ARC Centre of Excellence in Plants for Space Adelaide University, Waite Campus Urrbrae South Australia Australia; ^3^ Andy Thomas Centre for Space Resources Adelaide University Adelaide South Australia Australia; ^4^ International Flavour Research Centre University of Nottingham, Sutton Bonington Campus Loughborough UK; ^5^ International Flavour Research Centre (Adelaide) Adelaide University, Waite Campus Urrbrae South Australia Australia; ^6^ South Australian Research and Development Institute (SARDI), Waite Campus Urrbrae South Australia Australia; ^7^ School of Agriculture, Food and Wine Adelaide University, Waite Campus Urrbrae South Australia Australia

## Abstract

The current trend of individualized food items (nutrient content and flavor) can be particularly valuable to support people's health and well‐being in isolated environments where access to fresh foods and diverse nutrition is difficult. At the same time, bioactive compounds and micronutrients can be employed to countermeasure any health hazards characteristic for the respective environment. Outer space is well‐known as a hazardous environment due to microgravity, space radiation, and extreme living situation, whereas food monotony has led to insufficient caloric intake in the past. We are proposing to adapt the current trends of fortification and personalization for a novel space food system to support astronaut's health and increase food choice. Beverages as food matrix allow straightforward adjustment of their composition, whereas beverage emulsions as formulation strategy allow encapsulation of hydrophobic ingredients. This literature review gives an overview of food and beverage fortification on Earth, current and in‐development space food systems, common space health risks with the potential to be attenuated by nutrition/supplementation, as well as bioactive compounds potentially suitable for beverage fortification. Finally, a process concept is presented that could enable in‐space manufacturing of beverages for future space travel.

## 1 Introduction

The latest advances in space research and modern technology will enable pioneering and leading‐edge deep space projects in the close future. Whether it is the establishment of a space station in the lunar orbit or the first human on the Mars (NASA 2020)—in either case, astronauts will be facing significantly prolonged space missions compared to the ordinary time period of 6 months astronauts spend onboard the International Space Station (ISS) (NASA 2011). As space missions of current duration are already very demanding on both the astronaut's physical and mental health, increasing the duration of space missions will represent an even greater challenge to maintain astronaut's well‐being.

Health and well‐being cannot be sustained without a suitable nutrition that provides astronauts with energy and macro‐ and micronutrients required by their body for normal functioning in space. In the past of space exploration, space agencies struggled to provide an adequate diet to astronauts, resulting in the loss of 1%–5% of body mass, among others (Smith et al. 2015, 2019). Although the food items themselves seemed functional at providing nutrition (Gretebeck et al. 1994), their palatability, lack of choice, and lack of time were likely to discourage astronauts from eating sufficient amounts of foods (Smith et al. 2015, 2019; Cooper et al. 2011). These issues have since been overcome by easing food preparation, increasing quality and choice of foods, and providing more space dedicated for meal preparation and consumption in a communal setting (e.g., meal tables) (Smith et al. 2019). However, the space food system currently used for the ISS is unlikely to be adapted for long‐term space missions. To date, almost all foods consumed on the ISS are prepacked items of extensive shelf‐life at ambient temperature brought along from Earth (Bourland and Vogt 2010). The only exceptions are early‐stage studies on space farming during which astronauts are allowed to taste‐try their produce (Bunchek et al. 2024), as well as fresh vegetables and fruit brought by resupply vehicles (Douglas et al. 2020). Yet in both cases, quantities are too low to be considered an impact on the astronaut's nutritional status (Cooper et al. 2011). Bringing along all food items required on a 3‐year mission to Mars is not feasible, as their combined volume and mass would lead to an excessive fuel consumption while taking up too much room in the spacecraft (Perchonok et al. 2012). Furthermore, the requisite of shelf‐life for food items for a Mars mission is 5 years as safety measure, which is not yet technologically feasible, even when ignoring additional hazards such as long‐term exposure to space radiation (Douglas et al. 2020). It is unquestionable that novel space food systems will have at least one renewable component, that is, foods that can be grown, cultured, and/or produced in space. However, new food systems might revive nutritional issues observed in the past due to low palatability or acceptability. Therefore, palatability and acceptability need to be considered early‐on in the design of new space food systems.

An ideal space food system would provide practically endless choices of save, flavorsome, and nutritious meals, catering to different cuisines, cultural tastes, and personal preferences. Currently, astronauts are only allowed to choose around 20% of the food products to be consumed in space, with the remaining 80% being set by the mission planners (Douglas et al. 2020) in form of set meals that are repeated after 16 days (Cooper et al. 2011). Increasing food choice could help avoid menu fatigue and food monotony. An on‐demand production would reduce quantities of food items to be brought along, if renewable food sources and recycled water are utilized, which aligns with the approach of in‐space manufacturing (Bhundiya et al. 2022). This concept would translate to a more advanced version of the current in‐development 3D food printer, which can already produce different food textures using raw material, such as chocolate, dough, and starch (Lille et al. 2018; Mantihal et al. 2020; Pérez et al. 2019). We are intending to adopt this approach for personalized beverages due to their simpler food matrix, which also allows us to apply our expertise in liquid processing. Next to water, astronauts currently have access to a limited choice of reconstituted beverage, that is, pouches containing water‐soluble powders to recreate coffee, tea, and fruit‐flavored beverages (Cooper et al. 2011).

Reviewing current development in the beverage industry on Earth, next to an increased demand for personalized products, fortification of food items is popular (Kolb et al. 2014; Piorkowski and McClements 2014; Chen et al. 2023). Consumers intent to promote health, prevent disease, or to increase their physical and cognitive function by supplementing certain bioactive compounds, and fortified foods are perceived positively as long as taste and texture remain unchanged (Embling et al. 2024). At the same time, major health organizations like the WHO are recommending fortified foods as a convenient way to battle micronutrient deficiencies (van Schoor et al. 2024). The popularity of fortified food items mirrors the development of the personalized health approach in medicine. Personalized health supporters argue that the individualization of disease management and prevention based on personal circumstances, biomarker levels, and life styles is more effective than the current “one‐size‐fits‐all” treatment approach of patients (Snyderman 2012). The concept of fortification and personalized health might be beneficial for astronauts, as spending time in space is still accompanied by a range of health risks. Although the body reacts negatively to the changed environment, for example, low‐quality sleep is considered to be a response to changed daylight times, microgravity induces considerable, but partially reversible changes to the muscle system and skeleton, and radiation might lead to damage in the DNA that will only become evident in the long term in form of cancer (Afshinnekoo et al. 2020). Countermeasure studies have shown that the systematic consumption of certain compounds might balance these space‐induced health risks, although more research is required (Tanaka et al. 2017; Hellweg et al. 2020; Crucian et al. 2018). Therefore, fortification of certain space foods represents an option to consider when planning long‐term space travel, where fresh and diverse foods are more difficult to access.

Fortification of beverages can be implemented by simply dissolving hydrophilic compounds in the aqueous phase, whereas hydrophobic ingredients require a form of encapsulation, for example, emulsions. Emulsified beverages (also referred to as beverage emulsions) are systems containing very small amounts of dispersed oil (≤0.1 wt%) and are commonly used to add flavor to a beverage or to influence its appearance (Tan 2003). Current research also addresses the potential of beverage emulsion for fortification. However, high‐energy emulsification, as it is common in the food industry, is not convenient for space due to its high‐energy demands and likelihood to damage sensitive ingredients (Roohinejad et al. 2018). Alternative technologies, such as low‐energy emulsification processes and microfluidics, might present a solution for efficient in‐space production of fortified beverages.

Therefore, for this literature review, we are discussing a novel concept for a space beverage system that via appropriate formulation allows the incorporation of aroma and hydrophobic compounds. Appropriate process design allows personalization of the beverages’ composition in terms of flavor, sweetness level, and nutritional value as well as on‐demand production. For the concept design, inspiration is drawn from developments in the terrestrial beverage industry. To determine suitable hydrophobic compounds for fortification, current and novel space food systems, including the current approach to supplementation, are reviewed. For the process setup, suitable alternatives are sought to the commonly used processes in the beverage industry.

## 2 Space Health Risks

The everyday life in space, as realized by current technology, is vastly different from living on Earth. Due to the large distance to the Earth, the terrestrial gravitational force becomes so small that its effect is negligible, changing the behavior of fluids and objects in space, but has also considerate effects on the body. As there is no atmosphere around the spacecraft, it is exposed to significantly higher levels of cosmic radiation. The design of a spacecraft is resource‐efficient to save fuel, yet the implication is that the spacecraft provides little room for its crew. Considering these circumstances, NASA has defined the following space hazards: distance, confinement, hostile and closed environments, lack of gravity, and radiation (Afshinnekoo et al. 2020). On the basis of these environmental conditions, a variety of health risks emerge. In the following, three health implications are discussed, which have the potential to be mitigated by improved nutrition for long‐duration space missions.

### 2.1 Loss of Muscle and Bone

One of the major health concerns to be identified is muscle and bone loss (Smith et al. 2013). As modern humans spend a major part of the day in an upright position while doing activities like standing, sitting, and walking, the muscle system has adapted to counterweight gravity in the longitudinal direction of the body (Tanaka et al. 2017). Consequently, once this force diminishes in outer space, a share of the skeletal muscle—especially pronounced in the lower extremity—atrophies (Tanaka et al. 2017). A recent study observing the muscle volume of participants of a bed‐rest study is suggesting that the loss in muscles is stronger in women than in men (Trappe et al. 2023). Although muscles enable movement, the human skeleton provides a firm internal structure to bear and support the body weight (Tanaka et al. 2017; Stavnichuk et al. 2020). As the weight of the human body decreases due to the diminishing gravitational force, the bones react to the reduction in mechanical load by an increased rate of bone resorption (Smith et al. 2014; Stavnichuk et al. 2020). Next to increased bone resorption (up to 50%), studies have shown that calcium absorption during space flight is depleted by up to 50%, leading to an overall loss of approx. 250 mg bone calcium per day of space flight (Smith et al. 1999). An effective countermeasure for both muscle and bone loss has been found to be resistance exercise in combination with aerobic exercise (Smith et al. 2008, 2012). Currently, astronauts are required to perform 1.5–2 h of total exercise on 6 days per week (30–45 min of aerobic exercise and 60–75 min of resistance exercise) (English et al. 2020). However, the exercise regimen does not prevent bone resorption but rather stimulates the rate of bone formation so that the net bone density remains stable (Smith et al. 2008). When applied in the right way, astronauts even return from a space mission with an improved body composition, that is, increased lean body tissue and reduced body fat (Smith et al. 2012). Nonetheless, for the exercise regimen to work efficiently, an adequate dietary intake as well as vitamin D intake is mandatory (Smith et al. 2012).

### 2.2 Cell and DNA Damage

Damages to the astronaut's genetic material and body tissue are caused by increased oxidative and nitrosative stress as well as exposure to cosmic radiation during space travel (Afshinnekoo et al. 2020). Cell and DNA damage can lead to cell death, atrophy, inflammation, and cancer (Schumacher et al. 2021).

Reactive oxygen species (ROS, e.g., hydrogen peroxide, singlet oxygen, superoxide anion radicals, and hydroxyl radicals) and reactive nitrogen species (RNS, e.g., nitric oxide radicals, nitrogen dioxide radicals, and nitrous acid) are formed during regular cell metabolism in all living organisms (Hameister et al. 2020; Pizzino et al. 2017) and fulfill functions related to cellular signaling among other (Valko et al. 2007; Spitz et al. 2004). In healthy organisms, ROS and RNS are neutralized by enzymatic and nonenzymatic antioxidants (redox regulation) (Valko et al. 2007; Hameister et al. 2020). However, under the exposure to environmental stressor and/or lack of antioxidants as provided in nutrition, excess ROS and RNS damage cells and tissue, which is referred to as oxidative stress or nitrosative stress, respectively (Pizzino et al. 2017; Valko et al. 2007). Increased ROS and RNS have been found in astronauts during space missions caused by environmental stressors, such as microgravity and space radiation (Afshinnekoo et al. 2020; Goodwin and Christofidou‐Solomidou 2018; da Silveira et al. 2020).

While being jointly responsible for increasing oxidative and nitrosative stress, space radiation itself is also a reason for increased cell and DNA damage during space missions. The Earth is protected by its magnetosphere and its atmosphere from the majority of ionizing radiation prevalent in deep space, that is, electromagnetic and particle radiation (Montesinos et al. 2021). The sun emits occasionally solar particles during solar particle events, that is, solar flares and coronal mass ejections; however, they can be sufficiently deterred via spacecraft shielding (Nelson 2016). More concerning for human health is the constant exposure to galactic cosmic rays (GCRs), which are considered to be caused by supernova remnants (Blasi 2013). GCRs consists primarily of protons (85%) and helium nuclei (12%) and secondarily of electrons and positrons (2%) as well as high‐ and high‐charge particles (HZE, 1%) (Afshinnekoo et al. 2020; Durante and Cucinotta 2008). Due to the ISS's location in low Earth orbit, its crew is still partially protected against GCRs from the Earth's magnetosphere; however, astronauts are still exposed to elevated radiation (Cucinotta and Durante 2006). Long‐term radiation measurement onboard the ISS showed that the crew were exposed to radiation doses of approx. 0.7 mSv/day, yet radiation levels were dependent on solar activity and the ISS altitude (Berger et al. 2016, 2017, 2020). The badges, which every ISS crewmember wears to record personal radiation exposure, typically document a dose of 0.2–0.5 mSv/day (Shavers et al. 2024). Outside the low Earth orbit, radiation is higher (approx. 1–2 mSv/day for interplanetary travel and 0.5–1 mSv/day on the surface of planets) (Cucinotta and Durante 2006; Saganti et al. 2004), but long distances also extend the mission duration, leading in combination to considerably higher radiation doses for the space crew. For example, a 3‐year mission to Mars could accumulate to radiation doses of 1 Sv or higher per crew member (Wilson et al. 1995; Cucinotta and Durante 2006), yet the current career limit to cosmic radiation exposure is defined as 1 Sv by several space agencies (European Space Agency, ESA; Russian Space Agency, RSA; Canadian Space Agency, CSA) to keep the cancer risk within acceptable limits (Shavers et al. 2024). At the same time, long‐term exposure to GCR is underexplored and might lead to health consequences not yet foreseen (Cucinotta and Durante 2006; Cucinotta et al. 2013; Nelson 2016).

### 2.3 Space Anorexia

Space anorexia describes the tendency of astronauts to consume less food in space than suggested by the daily dietary recommendations designed for the mission (Da Silva et al. 2002). For example, in Smith et al. (2005) found after studying the dietary intake and the body composition of 11 astronauts before and after different prolonged space missions at the ISS that the astronauts ingested an energy intake reduced by 20% compared to the energy intake recommendations. The results align with nutritional data of earlier space missions, in which the astronauts’ energy intake was reduced by 30% or more in case of additional exercise (Smith et al. 2019). It was confirmed that the average reduction in caloric intake is not skewed by a few individuals who reduce their intake significantly but that it is rather a systemic problem, as groups of astronauts being together on the same mission tend to eat similar quantities of food (Wade et al. 2002).

Underconsumption of caloric requirements leads to a loss of body mass on Earth, and this causality is also true for space, as confirmed during the Apollo mission (Smith et al. 2015). In the case of space anorexia, loss of body mass has been reported to be on average 1%–5% regardless of duration of the flight or space agency (NASA, Roscosmos) (Smith et al. 2015, 2019). However, between individuals, the range in body mass loss is considerate, with some astronauts increasing body mass slightly and others losing up to 15% (Smith et al. 2019). Although some of the loss is caused by losing body water (Leach et al. 1996), it is negligible compared to the loss of muscle mass and fat tissue (Smith et al. 2015; Lane et al. 1998; Johnson et al. 1973). As the body is not provided with sufficient energy via food intake, it metabolizes fat tissue and proteins from both muscles (somatic proteins) and to lower extend organs (visceral proteins) as source of energy to maintain essential body function (Smith et al. 2019), whereas glycogen stored in the muscles is metabolized as source of glucose to maintain brain function, leading to a reduction in muscle volume (Smith et al. 2015). When metabolizing body protein due to undereating, synthesis of new protein is also decreased, which was confirmed for spaceflight during the MIR missions (Stein et al. 1999b). Both the metabolism of body protein and the decrease in body protein synthesis worsen the microgravity‐induced muscle loss and hinder the preservation of lean body mass during space flight (Smith et al. 2015). When astronauts are not meeting recommended energy intake, deficiencies in micronutrient supply are also likely due to the overall reduced intake of foods (Smith et al. 2019). Furthermore, underconsumption of food can worsen the impact of space flight on the immune system and the cardiovascular system (Crucian et al. 2018; Baran et al. 2021). Apart from health consequences on the individual astronaut, space anorexia may corrupt biochemical studies during space missions, as it remains unclear whether the observed effects are caused by space flight conditions (microgravity, space radiation, etc.) or space flight‐related malnutrition (Smith et al. 2015, 2019).

It is still unresolved why astronauts suffer from space anorexia (Da Silva et al. 2002). Unsuitability of space foods (regarding nutritional content only) can be excluded, as the consumption of space foods at recommended levels on Earth is able to maintain physical performance and body weight (Gretebeck et al. 1994). Moreover, under specific circumstances, astronauts demonstrated they were able to consume the required caloric intake. The Skylab mission, which included extensive metabolism studies, specifically required astronauts to meet a set diet on the basis of terrestrial energy and nutritional requirements, and astronauts managed to reach the nutritional targets (Rambaut et al. 1977). However, astronauts still suffered from a decrease in body mass, yet they predominantly lost lean body mass. The set diet was sufficient to meet energy requirements in space based on the retainment of the astronaut's adipose tissue (Rambaut et al. 1977). Even though movement in space requires less energy, overall energy expenditure does not change compared to Earth, likely due to increased resting metabolic rate and stress (Smith et al. 2019). The loss of lean body mass during the Skylab mission was instead attributed to the lack of gravity and associated loss of muscle and bone mass (Rambaut et al. 1977). Thus, a eucaloric diet as singular measure is not able to maintain body mass in general and lean body mass in specific (Smith et al. 2019). The Skylab mission was the first mission giving astronauts access to exercise equipment, that is, a cycle ergometer and a treadmill system; however exercise regimens were immature, as it took astronauts 10 days to understand the working principle of the cycle ergometer (Beyene 2004). Since then, knowledge about sufficient in‐space exercise regime has increased, and it was shown that applying a combination of aerobic and resistance training on almost every day in combination with consuming adequate caloric intake is able to maintain weight (Smith et al. 2015). Therefore, space anorexia is not caused by any systemic effect of life in space on the human body, as in principle, suitable food can be consumed in appropriate amounts to prevent loss of body mass when combined with appropriate exercise.

Questioning astronauts on their perceived appetite levels led to inconsistent results, with some even reporting an increased appetite (Smith et al. 2015; Agureev et al. 1997). A changed circadian rhythm caused by 180 min cycles of daylight and darkness on the ISS instead of 24 h day cycles (Afshinnekoo et al. 2020) in combination with the artificial light used inside the ISS (Brainard et al. 2013) might impact the hypothalamus and associated hormones, which regulates meal size and number (Varma et al. 2000). The changed circadian rhythm also greatly influences sleep quality, as astronauts on average only sleep 6 h instead of 8.5 h within a 24‐h period and report themselves to feel fatigued (Barger et al. 2014). A decreased appetite might also be related to the increased core body temperature in space, caused by the absence of gravity‐induced natural convection of air surrounding the body, which can impact physical and cognitive performance (Stahn et al. 2017). During space missions in the past, astronauts have reported that the time required for food preparation, consumption, and tidy up was too long to fit conveniently in a normal work day and hindered food intake (Smith et al. 2015). At the beginning of a space mission, 60%–80% of astronauts suffer from space motion sickness, with symptoms including cold sweating, loss of appetite, and vomiting (Heer and Paloski 2006). Space motion sickness is therefore a major contributor to space anorexia, and food intake remains reduced long after the symptoms subside, which is usually within the first week of space travel (Smith and Zwart 2008).

Palatability and menu fatigue, possibly in combination with a changed flavor perception, are considered another major contributor to space anorexia, as it has been shown that the palatability of foods and aroma perception has a significant influence on the appetite and caloric intake (Yeomans 1998; Boesveldt et al. 2018). Whether flavor perception changes during space flight remains unclear, see Section 3.1. Yet, food processing used for most space foods (dehydration and thermostabilization) is known to impact the organoleptic properties of foods negatively, for example, destroy temperature‐sensitive aroma compounds. Many space food items are stored in light‐weighted retour pouches. Shelf‐life testing of food stored in retort pouches showed the formation of off‐flavor and off‐colors in the foods and was assumed to be caused by Maillard and oxidation reactions (Catauro and Perchonok 2012). Additionally, some products are consumed directly from the pouches, preventing any visual sensory input of the food item. Yet, flavor perception is a multisensory sensation and can be impacted by appearance and color of the food, but also by its packaging and presentation (Motoki et al. 2023; Spence 2015). During crew debriefs, menu fatigue and lack of variety are often mentioned and let to the increase of the duration of the menu cycle from 8 to 16 days (Cooper et al. 2011). Other strategies against food monotony are providing a range of condiments and snacks (Smith et al. 2019). It is often suggested that the access to frozen food during the Skylab mission (the only space mission to date with access to frozen food), that is, higher palatability and variety of foods, has helped astronauts to consume the recommended caloric intake (Smith et al. 2015, 2019).

However, within recent years, the situation regarding underconsumption of foods and loss of body mass of crew onboard the ISS has significantly improved. Exercise regimens have been improved, and crewmembers consumed >90% of their energy intake recommendations, thereby maintaining body mass without losing muscle or bone mass (Smith et al. 2012, 2015). Increased food intake was accredited by providing more space on the space station assigned for food consumption, that is, a large galley table, and easing food preparation by installing a portable water dispenser, while increasing the diversity of available food items (Smith et al. 2019). Even though space anorexia seems to be resolved for missions on the ISS, novel space food systems designed for long‐term missions might lead to similar issues in the future. Understanding the reasons for underconsumption of foods and associated loss in body mass helps to prepare long‐term duration missions appropriately.

## 3 Space Foods and Space Nutrition

Space foods are all food products and food items designed for consumption by astronauts during space missions but also consider the food's packaging, preparation, and disposal (Bourland and Vogt 2010; Oluwafemi et al. 2018). Space nutrition describes the approach of providing astronauts with all nutrients required for maintaining health and metabolism in space (Oluwafemi et al. 2018). Nutrition and space foods have been an integral part of the design and planning of space missions from the very beginning. Already on the second crewed space mission in 1961, Soviet cosmonaut Gherman Titov tested whether eating in space was possible, dismissing concerns that swallowing foods in space was impossible due to microgravity (Bourland and Vogt 2010). Although early‐stage space foods consisted of lightweight and compact snack‐like foods, such as bite‐sized cubes, freeze‐dried powders, and semi‐liquids (Dakkumadugula et al. 2023; Perchonok and Bourland 2002), with increase of the duration of space missions from a few hours to 4–6 months space foods and nutrition became more varied and diverse. The following sections explore current and anticipated approaches to nutrition and food systems in space.

### 3.1 Flavor Perception in Space

Flavor perception during food consumption consists of two key sensory experiences: The taste (sweet, salty, sour, umami, and bitter) as perceived by the taste receptor cells located in taste buds on the tongue and the aroma as perceived by olfactory receptor neurons in both the nasal cavity (orthonasal olfaction before food consumption) and in the retronasal passage (retronasal olfaction during and after consumption). The aroma consists of volatile aromatic compounds with odor properties when present above the odor detection threshold, that is, the concentration (typically in ppb or ppm) at which the compound's odor is detectable for most humans. However, both taste and flavor perception can be very individual. Taste perception depends on health status, age, gender, ethnicity, genetics, and taste phenotypes (Yang et al. 2020). Taste phenotypes are mainly nontasters/supertasters, thermal tasters, and sweet likers (Yang et al. 2020). Nontasters and supertasters describe individuals that are less or more sensitive to taste stimuli, respectively, compared to medium tasters, often tested via the bitterant 6‐*n*‐propylthiouracil (PROP) (Pickering et al. 2013). Thermal tasters can perceive a taste sensation induced only via temperature (Skinner et al. 2018), for example, hot water can induce a sweet taste perception in some thermal tasters. For individuals that are sweet likers or sweet dislikers, culinary delight increases or decreases with increasing sweetness level, respectively (Garneau et al. 2018). Regarding aroma perception, it is estimated that approx. 20% of the population suffer from an olfactory disfunction, which can present in the reduction of aroma perception (hyposmia), the absence of aroma perception in general or of specific compounds (full or partial anosmia), and the presence of aroma perception in the absence of aroma stimuli (phantosmia) (Hummel et al. 2023). In addition, individual oral microbiota and saliva composition influence the release of aroma compounds from the food matrix (Parker et al. 2020). Flavor perception can further be shaped by temperature, texture, color, and trigeminal effects (e.g., cooling sensation induced by menthol), among others. Therefore, flavor perception, as studied on Earth, can greatly vary between individuals, and flavor studies typically require a considerable number of participants to be able to draw conclusions.

When looking at flavor perception in space, the current state of knowledge is inconclusive. Early studies on changes in the nervous system during the Challenger and Skylab missions could not detect a change in taste threshold or olfaction (Watt et al. 1985; Smith et al. 2019). Outcomes of studies on sense alteration in general have been contradicting each other due to considerate changes in study design over the years, low numbers of people that have travelled to space to date, but also low numbers of participants joining specific studies (typically less than 6), who have not been trained for flavor analysis (Gonzalez Viejo et al. 2024; Taylor et al. 2020). Yet, in anecdotal reports and crew debriefs, a change in flavor perception in space has been repeatedly mentioned (Smith et al. 2019; Douglas et al. 2016), although not experienced by every astronaut (Taylor et al. 2020). Those that have experienced changes reported that their food likings and preferences changed during space missions, even though liking and acceptance of provided food items had been extensively tested pre‐flight (Douglas et al. 2016). Head‐down bed rest studies are currently considered the best way of simulating the effect of microgravity on the human body, yet head‐down bed rest studies on flavor perception have reported contradictory results as well (Gonzalez Viejo et al. 2024; Smith et al. 2019). Still, there have been two main mechanisms proposed that are likely to influence flavor perception during space flight.

- Shift in fluids: Due to microgravity, the distribution of fluids in the body changes, leading to a higher fluid content in the head compared to pre‐flight. Especially during the first days or weeks, the higher fluid content leads to closed nasal cavities (Yakovleva and Baranova 1982), impacting aroma perception (Parker et al. 2020), and is often accompanied by space motion sickness, which leads to a decrease in appetite in general among other symptoms (Heer and Paloski 2006). Although the body adapts to the shift in fluids and nasal congestion resolves, nasal cavities might still be impacted by the fluid shift, which could change odor perception (Taylor et al. 2020).
- Spaceship environment and confinement: The spaceship environment is a highly regulated, confined space to ensure astronaut's survival in a place where human existence would otherwise not be possible. As there is no natural source of water in outer space, and natural sources of water on moons and planets are still to be accessed, water supply in spacecrafts is achieved via water recycling. Even though water recycling, which is coupled with an air purification system, considers removal of volatile organic compounds from air and water (Carter et al. 2008), trace amounts might still remain in both water and air, which could change the taste of food rehydrated with recycled water and general odor perception, respectively. However, comparing the reported composition of recycled water and ambient air with odor threshold values, an influence of recycled water and ambient air on flavor perception has been considered unlikely, although the presence of certain aldehydes above odor threshold levels in the ambient air should be further investigated (Taylor et al. 2020). Additionally, air moves differently in microgravity due to the absence of gravity‐induced natural convection (Sielaff et al. 2022), which might impact the transport of aroma compounds to the nasal cavities prior to consumption. Furthermore, research on Earth showed that exposure to elevated levels of noise influences flavor perception negatively, being one of the factors for passenger dissatisfaction of food during airplane travel (Spence 2017). The noise levels onboard the ISS are elevated (Limardo et al. 2021), but its impact on food perception, especially over long periods, needs to be further investigated (Taylor et al. 2020). Finally, the unique spacecraft environment imposes a reality of social isolation and spatial confinement for the astronauts, which results in combination with their high workloads to increased levels of stress (Radstake et al. 2022; Arone et al. 2021; Yin et al. 2023). On Earth studies have suggested an influence of mental health on flavor perception (Ileri‐Gurel et al. 2013), thus, individual reports about changed flavor perception might be influenced by personal perception of stress levels.

### 3.2 Current Space Food System

In space nutrition, one of the first questions addressed was whether energy requirements during space flight change. It has since been concluded that the in‐flight energy expenditure in 0 g is approximately the same as in 1 g (Lane et al. 1997, 1998). Even though less muscle activity is required to move in weightlessness, it is likely that increased stress or metabolic response is increasing the energy expenditures, leading to an overall unchanged energy requirement compared to pre‐flight (Smith et al. 2019). However, when exercising intensively during the space mission, energy expenditure can exceed pre‐flight requirements (Stein et al. 1999a). For space missions, a eucaloric diet is intended, that is, the number of calories spent on a daily basis is supposed to match the number of calories consumed (Smith et al. 2015). Therefore, energy requirements are estimated individually considering body weight, age, height, and daily resistance exercise using the energy intake estimation equations provided by either WHO (moderate activity correction) for ISS missions (World Health Organization 1985; Smith et al. 2019) or by the National Academy of Medicine (USA) for NASA exploration missions of extended duration (*d* < 365 days) (Smith et al. 2019; Smith and Zwart 2020; Trumbo et al. 2002). For extravehicular activity, for example, space walks, an additional 200 kcal/h is added to the recommended energy intake of the day (Smith and Zwart 2020). The energy intake is recommended to be distributed in healthy ratios among the consumed macronutrients, that is, 50%–55% carbohydrates, 25%–35% fat, and 0.8 g/kg body weight protein (not exceeding 35%; 2/3 animal protein and 1/3 vegetable protein) (Smith et al. 2019; Smith and Zwart 2020), and these specifications will also be adapted for long‐duration space missions (Cooper et al. 2011). Requirements of macronutrient and micronutrient intake have been based on terrestrial nutrition research and optimized with the experience obtained from space missions. Required energy and nutrient intake is estimated prior to the mission, and appropriate foods in the form of three meals per day and additional snacks are provided (Bychkov et al. 2021), yet it is recommended to supply the space crew with excess foods to enable choice in meals and thereby facilitate adequate food intake (Smith and Zwart 2020).

**FIGURE 1:**
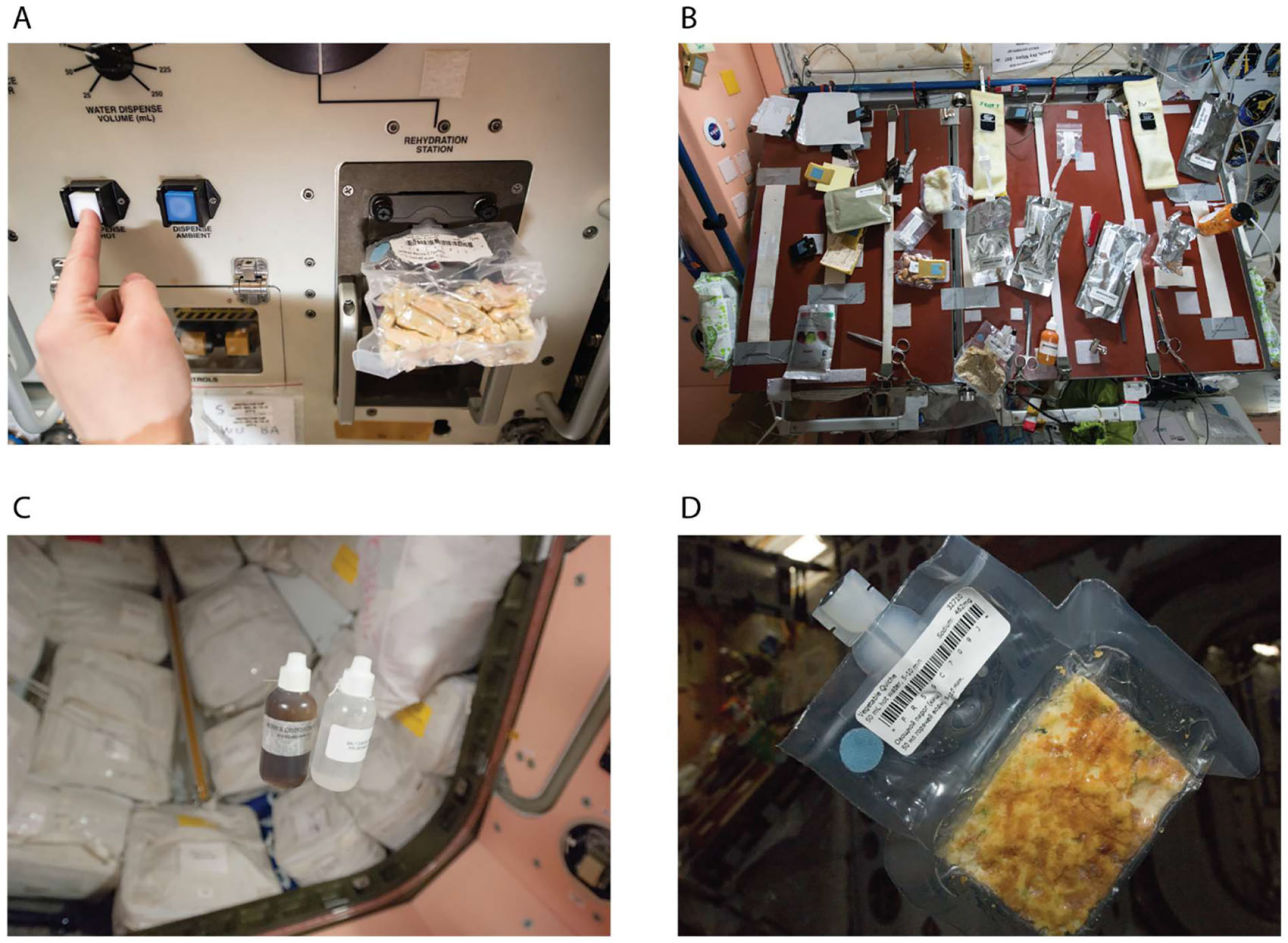
(A) Food rehydration station for the preparation of dehydrated space food items; (B) meal table in the Russian module Zvezda (ISS) with various food items and Velcro straps to secure food items; (C) dispenser for the liquid formulation of pepper and salt; and (D) thermostabilized vegetable quiche. Courtesy of NASA.

Currently, the space food systems consist entirely of prepacked food items that are brought from Earth to provide nutrition for the whole duration of the space mission (see Figure 1A–D). In the case of the ISS, restocking of foods several times a year is achieved via resupply vehicles (Douglas et al. 2020). However, selecting adequate foods for space exploration is not simple. Many food items have proven to be hazardous in space, and therefore foods and associated space food systems need to fulfill a range of requirements prior to be allowed on spacecrafts. Food items and appliances for their preparation must be safe, stable, palatable, nutritious, reliable, diverse, user‐friendly, and resource‐efficient, ensuring that the provision of foods is maintained, while minimizing the hurdles preventing astronauts from adequate food consumption (e.g., complicated food preparation, lack of taste, and food monotony) (Douglas et al. 2020). Yet, these requirements reduce the variety of foods that can be taken to space drastically compared to foods available on Earth. Fresh foods (fruit, vegetables, meats, fish, and dairy products) do not provide sufficient shelf lives, and their microbial load could cause contamination of the spacecraft (Pierson et al. 2011), and the accidental consumption of spoiled food can cause food poisoning (Douglas et al. 2020). Freeze‐dried powders, although having long shelf life, turned out to be unsuitable for space travel due to difficulties in properly rehydrating the powders, and the risk of emitting crumbs or particles to float and move freely in the spacecraft, which could damage equipment (Perchonok and Bourland 2002; Bourland 1993). The risk of crumb or particle formation also prohibits bread or related food items from space travel, whereas pepper and salt are provided in liquefied form (Bourland and Vogt 2010). Although freezers and refrigerators, which enable long shelf lives of stored foods, have been successfully established onboard the ISS for biological and medical studies, their resource requirements (energy, space/volume, and fuel) for food storage only are considered not feasible (Smith et al. 2019). Therefore, all food items need to be designed to provide the required shelf life of 18 months at ambient temperature (Cooper et al. 2011).

**FIGURE 2:**
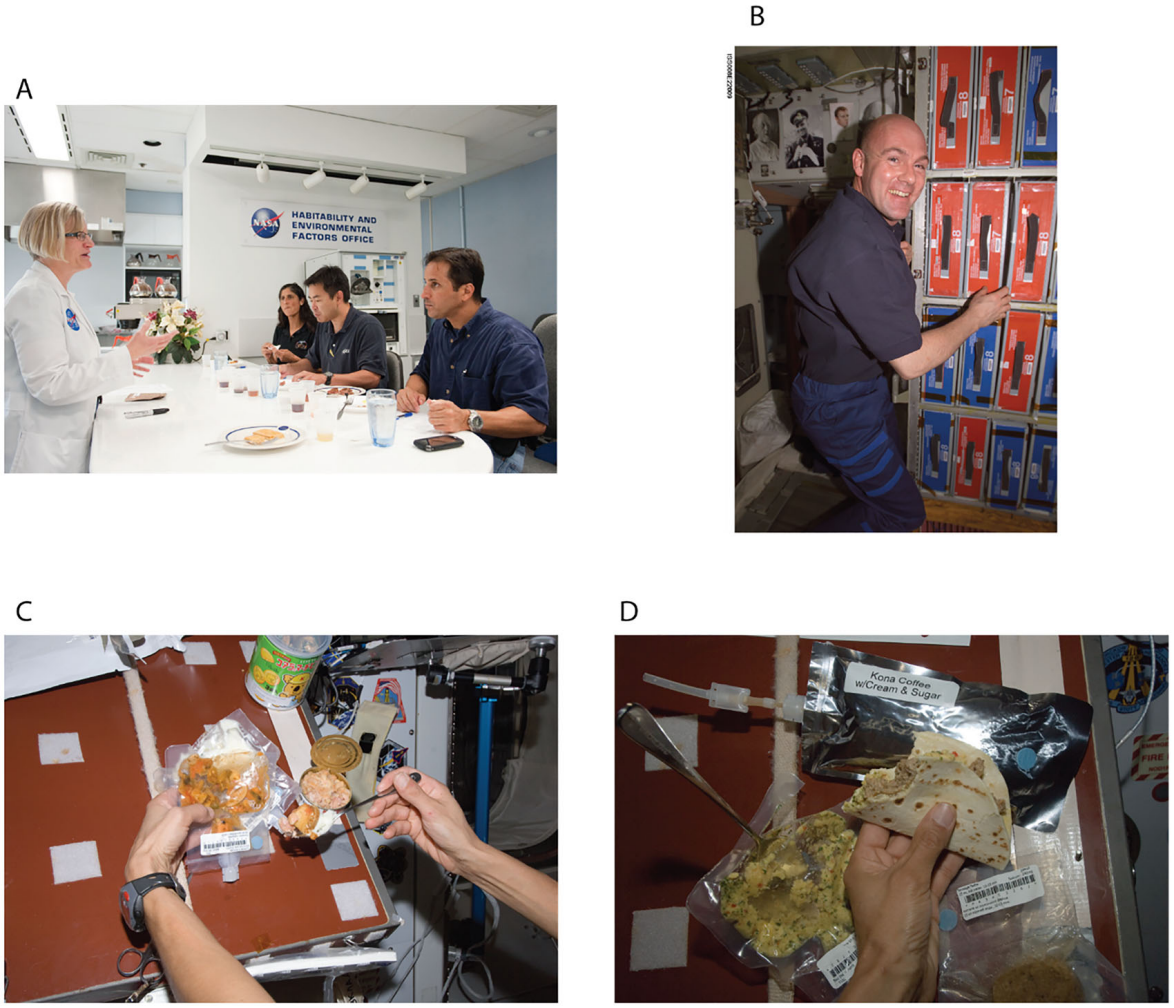
(A) Astronauts Sunita Williams, Akihiko Hoshide, and Joe Acaba during their pre‐mission food tasting session; (B) Astronaut Andre Kuipers in front of the food storage lockers in the Russian Module Zvezda (ISS); and (C and D) different space food items are prepared for a meal, for example, canned food, thermostabilized food, extended shelf‐live bread (tortilla), and a coffee pouch. Courtesy of NASA.

To achieve suitable stability and reduce microbial load and enzymatic activity, foods are treated via thermostabilization, radiation, or dehydration (Cooper et al. 2011). Thermostabilized and irradiated food can simply be reheated prior to consumption in a convection oven in the spacecraft, and dehydrated food is rehydrated with a rehydration station that provides hot water (Cooper et al. 2011; Smith et al. 2019; Bourland and Vogt 2010). Although dehydrated food items have the advantage of being lightweight due to the removed water, recycled water resources onboard the ISS are limited, and mission planners are careful not to overly rely on dehydrated food items (Bourland and Vogt 2010). Yet, all beverages provided (coffee, tea, lemonade, and fruit‐flavored drinks) are dehydrated powders in vacuum‐sealed beverage pouches (Cooper et al. 2011). Other reliable space foods are so‐called natural‐form foods and extended shelf‐life bread products. Natural‐form foods are common, commercially available foods of low‐ or intermediate moisture content, which enables a long shelf life, such as nuts and dried fruits (Cooper et al. 2011). Extended shelf‐life bread products are non‐crumbling, common bread products such as tortillas, waffles, and bread rolls, which have been adapted to provide a longer shelf life than their commercially available counterparts (Cooper et al. 2011). Of the space‐proven foods, astronauts are only allowed to choose around 20% of the food products to be consumed in space, the rest is set by the mission planners (Douglas et al. 2020), who design a menu cycle of 16 days (Cooper et al. 2011) (see Figure 2A–D). All space food items are specifically packaged to extend their shelf‐life, that is, protect foods against exposure to light, moisture, oxygen, and microorganisms (Cooper et al. 2011; Smith et al. 2019). Currently, fresh fruits and vegetables are in fact available for the crew on the ISS via the frequent resupply; however, they need to be consumed within the first days after arrival to prevent spoilage (Bourland and Vogt 2010; Smith et al. 2019). Due to their limited availability, the primary intention of providing fresh fruits and vegetables is to support the psychological state of the crew instead of nutrition (Cooper et al. 2011).

### 3.3 Current Strategy of Supplementing Nutrients in Space Exploration

Currently, the only food supplement provided during space missions is vitamin D (Smith et al. 2019; Smith and Zwart 2020). On Earth, the main natural source of vitamin D is exposure to ultraviolet B light (UVB), which enables the formation of vitamin D3 via cutaneous synthesis from precursors in the skin (Raymond‐Lezman and Riskin 2023). Vitamin D3‐rich foods, such as sea fish and related products as well as eggs, contribute to lesser extent to the vitamin D supply (Benedik 2022). Regardless of source, vitamin D3 requires to undergo hepatic and renal activation in liver and kidney, respectively, to become biologically active. In space, however, the crew is not only protected from UVB light via shielding (Zeitlin 2021), but foods rich in vitamin D3 are also not easily available in space. Space agencies have therefore relied on supplementation of vitamin D3 in form of tablets (Zwart et al. 2009), ensuring sufficient absorption of the lipophilic micronutrient by requesting astronauts to consume 30%–35% of total daily calories in form of fat during ISS missions (Smith et al. 2019). Although initial supplementation doses did not maintain sufficiently vitamin D levels in the blood, increasing the vitamin supplementation to 800 international units per day (20 µg/day) for ISS missions and to 1000 international units per day (25 µg/day) for NASA exploration missions helped maintaining adequate vitamin D levels during space missions even at suboptimal diet (Smith and Zwart 2020; Smith et al. 2012, 2019). Additionally, the vitamin D3 supplementation tablets remained sufficiently stable over longer periods in space (Zwart et al. 2009).

Although malnutrition is a common health issue in space, NASA does currently not recommend nutrient supplementation other than vitamin D (Smith and Zwart 2008). The present strategy of space nutrition is to supply astronauts with all macro‐ and micronutrients via the food items available during space missions (Smith and Zwart 2020). Space nutrition is intended to be as healthy as possible, as an unhealthy diet cannot simply be counterbalanced by micronutrient supplementation (Zwart et al. 2021). Natural and standard foods provide many advantages over supplements, for example, a complex composition of hundreds of different compounds that provide flavor, nutrition, dietary fiber, and positive health effects (Smith et al. 2019; Brandt et al. 2004; Smith and Zwart 2020). The degradation of nutrients in natural and standard foods before or during space mission due to preservation techniques or space radiation is a concern, yet nutrients in foods stored for 596 days on the ISS showed no faster degradation than on Earth (Zwart et al. 2009). Still, the issue requires further investigation, especially for space missions lasting a few years and leading outside the Earth's magnetosphere. Alternative approaches to prevent long‐term nutrient degradation in long‐term shelf‐life foods is the development of in‐space farmed foods (see Section 3.4).

Additionally to the nutritional value, the experience of enjoying familiar foods in a social setting during mealtimes can provide psychological support (Smith et al. 2019; Smith and Zwart 2020). Thus, replacing foods with supplements is not possible. Increasing the number of supplemented micronutrients, for example, those that are identified as deficient from blood and urine diagnostics, is not advisable. In those cases, the nutrient deficiency is caused by underconsumption of food, therefore, measures should be taken to improve food consumption and uptake in the first place (Smith et al. 2019). Taking supplements to treat malnutrition instead of improving the food uptake might also trigger astronauts into believing inadequate food consumption can easily be balanced with supplements, even though their nutrient bioavailability and metabolization can differ compared to foods, thereby potentially worsening individual food intake (Smith et al. 2019). Supplementing a micronutrient or bioactive compound is only recommended when the compound is essential but cannot be provided adequately via space foods or when the supplementation of the compound is proven to provide a positive health effect that is otherwise not obtainable (Smith et al. 2019). Thus, any supplement added to the daily food nutrition needs to have proper scientific proof to enhance health and well‐being in a way that could not be achieved via space foods alone.

### 3.4 Novel Space Food Systems

Novel space food systems are developed and designed for long‐term space missions in particular, such as the first crewed mission to Mars, the closest planet to Earth. A return mission to Mars is predicted to take 2.5–3 years (Konstantinov and Petukhov 2012; Douglas et al. 2020), that is, 6 months to cover the distance between Earth and Mars, and at least 18 months of research and exploration on Mars itself (Douglas et al. 2020). Accompanying food systems (equipment, food items, and ingredients) are required to maintain working conditions, palatability, and nutrition under space conditions for 5 years (Douglas et al. 2020). The current space food system, that is, bringing thermostabilized and dehydrated foods with frequent restocking of fresh food items from Earth, is not suitable for various reasons. Restocking at the rate of the ISS (approx. every 6 months) during a Mars mission is not realistic, although scenarios are discussed where food is prepositioned on the Mars prior to the arrival of the crew to allow restocking (Cooper et al. 2011). Furthermore, the volume of prepackaged food required to ensure human survival of a small crew over 3 years would require too much storage room in the spacecraft to be feasible (Cooper et al. 2011). Finally, current space food items are also lacking the required stability, as they have a shelf life of 18 months only (Cooper et al. 2011), and deteriorate beyond that time frame (Cooper et al. 2017). It is also unclear how long‐term exposure of cosmic radiation will impact nutritional content and food quality (Watkins et al. 2022). However, in general, there has been yet no food system identified that could last for 5 years without any failure (deterioration of equipment, spoilage of food, and loss of nutrients, etc.) (Douglas et al. 2020). It is likely that future long‐term missions apply different space food systems that work in synergy but also ensure provision of food in the worst case scenario of one system failing. After all, the failing of all space food systems, that is, no provision of food for the space crew, would have catastrophic consequences for the mission, as there is no option of a quick emergency return (Douglas et al. 2020). A likely synergy is to complement the diet of prepacked foot items or food ingredients with regenerative systems, that is, systems that produce fresh food during the space mission (Perchonok et al. 2012). Regenerative systems are intended to be resource‐efficient, circular systems, for example, producing food from waste and using recycled water. Not only allow regenerative systems to decrease the load of foods to be brought along, but also fresh foods can provide nutrients that have depleted in prepacked foods during their production or storage (Perchonok et al. 2012). Current research focuses mostly on regenerative systems applying farming techniques and microorganism, including algae and insects for food generation.

**FIGURE 3:**
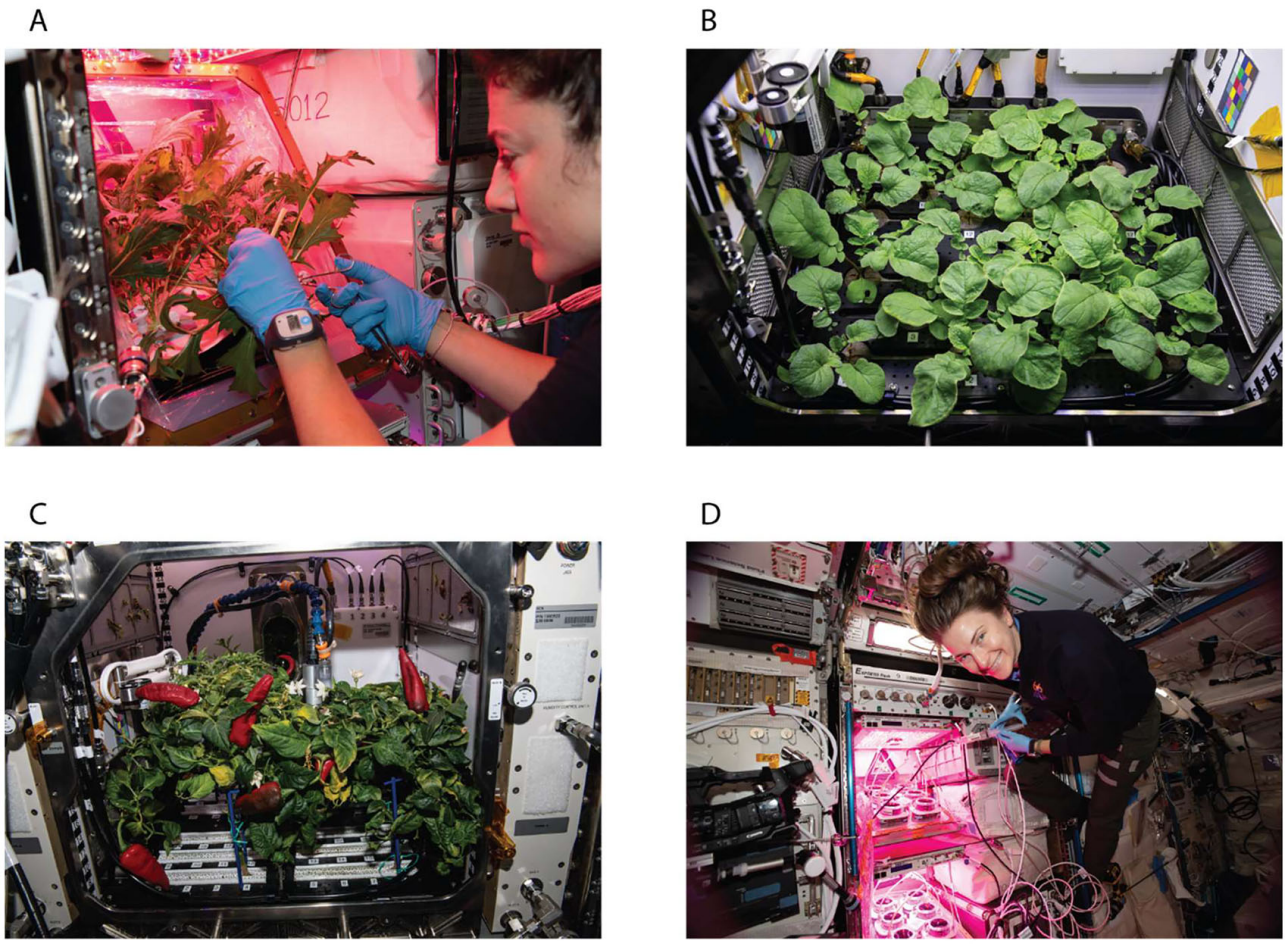
(A) Astronaut Jessica Meir harvests Mizuna mustard leaves grown in the Veggie greenhouse; (B) radish plants in the Advanced Plant Habitat; (C) chile peppers in the Advanced Plant Habitat; and (D) Astronaut Kayla Barron performs work on the Veggie greenhouse. Courtesy of NASA.

**FIGURE 4:**
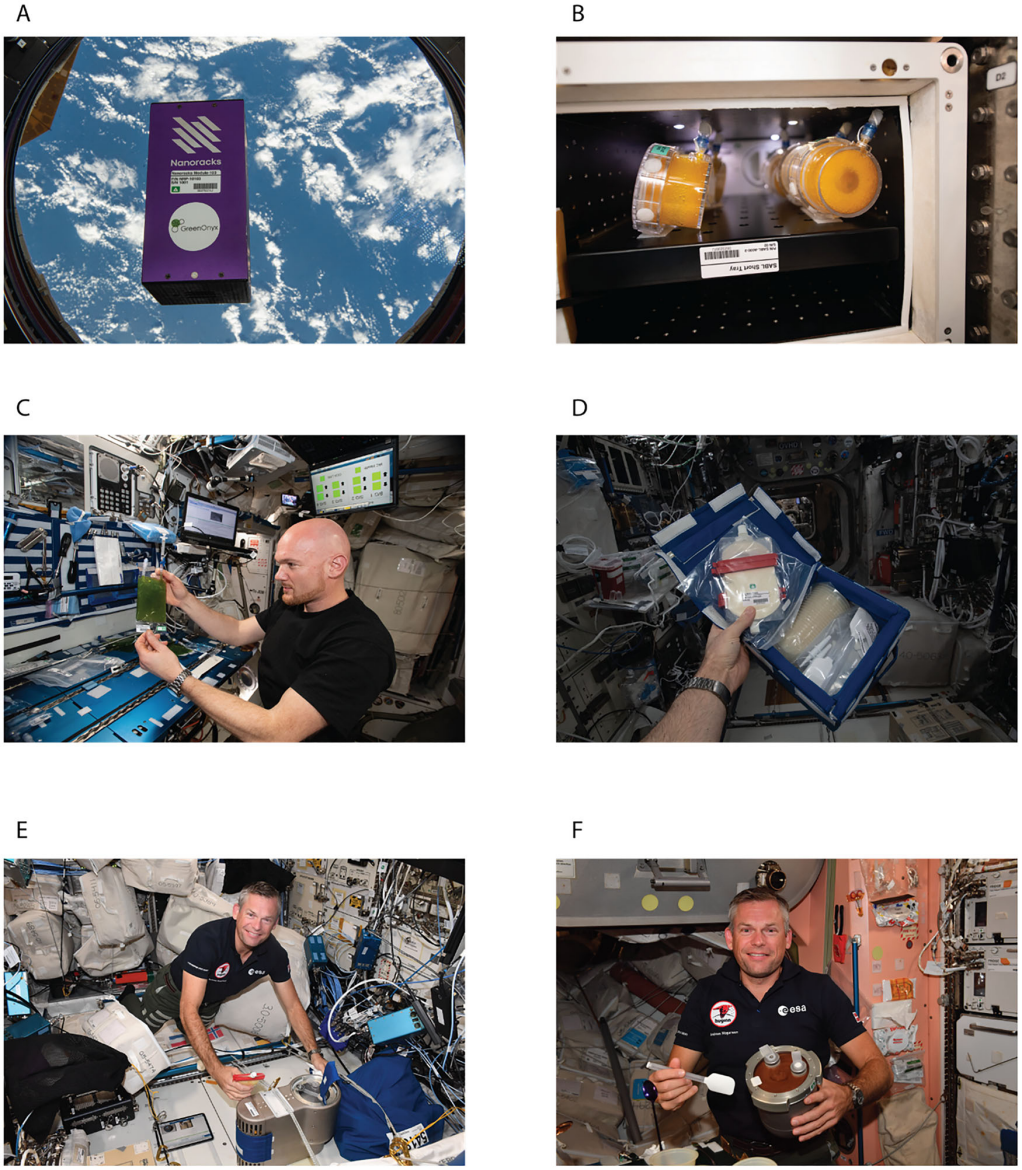
(A) Duckweed grown inside the Nano Growth Module (GreenOnyx); (B) in‐space cultured microbes of the BioNutrients project within the Space Automated Bioproduct Laboratory (SABL) of the ISS; (C) Astronaut Alexander Gerst holds a culture bag containing in‐space grown algae; (D) the Food Processor Consumables Kit containing edible foam made from the natural polymer PHA; (E) Astronaut Andreas Mogensen next to the Food Processor from the French Space Agency, which investigates more advanced food preparation in space; and (F) Astronaut Andreas Mogensen holding chocolate mousse made in‐space with the Food Processor; (A–D) Courtesy of NASA; (E and F) Courtesy of ESA.

- Space farming: Investigating plant growth and farming in microgravity for food production in space is currently the most popular approach, and early stages are already applied on board of the ISS (see Figure 3A–D). NASA's pick‐and‐eat Vegetable Production System (Veggie), a benchtop‐size greenhouse, has been growing leafy greens since 2014, for example, most recently mizuna mustard greens (Bunchek et al. 2024). The Advanced Plant Habitat (APH) is the closed‐loop, automated equivalent to Veggie on the ISS, and has been used to grow wheat among other (Monje et al. 2020; Nguyen et al. 2023). In the close future, Ohalo III will be established on the ISS as first larger scale crop production growth chamber (Poulet et al. 2022). Identifying crops suitable for space (fast growing, nutrient dense, and resilient to stress, etc.) led to unconventional suggestions, for example, the aquatic plant family Lemnaceae, colloquially known as duckweeds (Polutchko et al. 2022) (see Figure 4A). Vertical farming, engineered growing media, hydroponics, and aeroponics are discussed as potential farming techniques (Morrow et al. 2017). Combining hydroponics with fish farming (aquaponics) would provide astronauts with an additional source of food while reducing the need for fertilizer (Kitaya et al. 2023).
- Microorganisms: Microorganisms require little resources and can even utilize waste accumulating during space travel, while growing considerably faster than plants, and sensory characteristics as well as the nutritional profile can be adapted via gene engineering (Llorente et al. 2022). Promising microorganisms for space travel are yeasts and algae (see Figure 4B,C), specifically cyanobacteria producing spirulina (Llorente et al. 2022; Fais et al. 2022; Vinayak 2022; Walker and Granjou 2017), and is part of the European Space Agency's approach to a circular life support system called MELiSSA (Micro‐Ecological Life Support System Alternative) (Walker and Granjou 2017).
- Insects: Similarly to microorganisms, insects can utilize waste products for growth, but also inedible biomass from space farming, and are therefore considered suitable for space travel (Katayama et al. 2009; Kok and van Huis 2021; Tong et al. 2011). Compared to other animals, insects require less space and resources, produce less waste in form of excrement or inedible sections, and less or no noise (Tong et al. 2011). Silkworms, for example, offer a suitable nutritional profile of nutritional profile in combination with many micronutrients, while producing silk fibers as byproduct (Tong et al. 2011).

However, there is currently little research on developing novel space food processing technologies, which would aid food preparation from food ingredients, or processing of grown crops, microorganisms, and insects, as existing food processing technologies on Earth need to be adapted to environments of low gravity and air pressure (Perchonok et al. 2012). The French Space Agency is currently developing a Food Processor that aims to give astronauts access to a range of simple cooking functions, similar to modern cooking appliances (see Figure 4D–F), but unfortunately no official publication could be found outside the public image database of the European Space Agency (2023a, 2023b). During analog missions, that is, crewed missions in enclosed habitats on Earth mimicking spacecraft and space missions (Reagan et al. 2012), it became evident that giving complete autonomy to astronauts in relation to their food choice resulted in macro‐ and micronutrient deficiencies and loss in body mass, even though the supplied food system would have been able to provide sufficient nutrition (Zwart et al. 2021). Therefore, future long‐term missions will require ground‐based support to facilitate meal planning and ensure an appropriate nutrition.

Apart from the current developments in regenerative space food systems, many food companies take an interest in developing novelty foods for space or in supporting space food research. Yakult is partnering with the Japanese Aerospace Exploration Agency (JAXA) to investigate the feasibility and effect of probiotics for new space foods (Sakai et al. 2018; Yakult Honsha Co. 2019). Argotec and Lavazza have collaborated to design the first espresso machine for the ISS (ISSpresso) (Di Tana and Hall 2015). The Australian 4 Pines Brewing Company, together with Saber Astronautics, has developed a stout incl. adapted beer bottle that aims to enable beer consumption during private space tourism (Howell 2018). The start‐up Bake in Space has developed a crumb‐free bread and an accompanying oven, allowing baking and bread consumption in space (Ceurstemont 2017).

## 4 Suggested Technology for In‐Space Manufacturing of Fortified, Personalized Beverages

In the following section, technological options are presented to enable the in‐space manufacturing of fortified beverages containing micronutrients or bioactive compounds encapsulated in a beverage emulsion. The spacecraft environment is a unique place, as (1) availability of energy is limited, (2) physical space inside the spacecraft is limited, and (3) any additional mass increases fuel consumption.

### 4.1 Advances in the Terrestrial Nonalcoholic Beverage Industry

Nonalcoholic beverages are of particular interest as food item category to consider for future space food systems due to their popularity worldwide (Piorkowski and McClements 2014) in combination with a history in food innovation. Since the invention of the modern carbonated soft drinks’ precursors in the 19th century (Wolf et al. 2008), new beverage products have been constantly emerging to meet upcoming tastes and preferences of consumers on Earth. For instance, within the last decades and the upcoming of high obesity rates in both industrialized and developing countries, its citizens have been becoming increasingly health conscious (Kotilainen et al. 2006), creating a demand for beverages that are low‐caloric, thus leading to the development of new products such as sugar‐reduced soft drinks and flavored waters and an increase in bottled water sales (Piorkowski and McClements 2014). Aligning with the trend for fortified and functional foods, beverages have been regarded as one of the foods best fit for fortification, as there are a range of appealing probiotics, micronutrients, and bioactive compounds suitable to be added to a liquid system, whereas the beverage's formulation, package, and look can be adapted to the consumer's preferences (convenience for the consumer), and a long shelf live simplifies transport and storage (convenience for the distributer) (Corbo et al. 2014). The long shelf‐life and versatility for food fortification make beverages a well‐suited food matrix for supplementation in space. Examples for commercial product groups of fortified drinks containing functional ingredients are as follows:

- Probiotic drinks: The first functional foods were yogurt‐based beverages enriched with probiotics in Japan in the 1930s (Puiggròs et al. 2017; Yakult Honsha Co. 2019). Since then, the majority of probiotic drinks are still dairy‐based drinks, although alternatives based on soy bean and coconut have been developed as vegan option, and there is interest to fortify fruit juices with probiotics (Naseem et al. 2023).
- Plant‐based milk alternatives: plant‐based (soy bean, coconut, almond, cashews, oats, etc.) dairy alternative beverages have increased considerably in popularity in Western countries and are typically fortified with calcium, vitamin D, and vitamin B12 to draw closer to the nutritional profile of dairy products (Craig and Fresan 2021).
- Sport drinks: Sport drinks are formulated to provide hydration, carbohydrates such as glucose, fructose, and sucrose as easily accessible energy source, and essential electrolytes such as sodium, potassium, calcium, and magnesium before and during exercise (Corbo et al. 2014; Gupta et al. 2023). Vitamin B and botanical extracts such as ginseng, guarana, acai, ginkgo, and yerba mate are commonly added as functional ingredients (Gupta et al. 2023).
- Energy drinks: Energy drinks are formulated to provide an “energy boost,” that is, counteract effects of tiredness and exhaustion on cognitive function, alertness, and endurance (Gupta et al. 2023; Raizel et al. 2019). Next to sugars as energy source, they contain stimulants, micronutrients, and herbal compounds as functional ingredients, such as caffeine, guarana, vitamin B, ginseng, yerba mate, and ginkgo (Gupta et al. 2023; Raizel et al. 2019).

However, not all fortified beverages can be considered a functional beverage, as they do not fulfill the requirements defined above. Consuming beverages with high sugar content, such as sport and energy drinks, regularly does not promote optimal health and is even connected with a range of health risks (obesity, diabetes mellitus, nonalcoholic fatty liver disease, cavities, among others) (Kregiel 2015). Other ingredients of fortified beverages can also have negative long‐term effects, for example, enamel corrosion caused by the drink's acidity (Brima and Abbas 2014), as well as the overall formulation, for example, a strong correlation of poor mental health and regular consumption of energy drinks in adolescents (Masengo et al. 2020). These findings need to be considered when developing the overall composition of beverages for space application, as these are intended to support optimal health in space instead of cause adverse health effects.

Another trend in the beverage industry that emerged recently is the consumer's demand for individualization (Kolb et al. 2014). Due to the increased request for the optimization of health, well‐being, and performance, consumers want their beverages to fit to their individual needs, but consumers also like their beverages to align with preferences in appearance and taste. The option for personalization is valuable for novel space food systems, as it can enhance the astronauts’ acceptance as an adaption to personal preferences is possible. Responding to the customers’ interest on Earth, beverage companies like Coca‐Cola and PepsiCo developed beverages dispensing systems that allowed different levels of personalization.

Both The Coca‐Cola Company and PepsiCo developed a line of novel beverage dispensers for commercial use: *Coca‐Cola Freestyle* and *Pepsi Spire*, respectively. The *Coca‐Cola Freestyle* relies on micro‐dispersing technology to formulate more than 150 beverages on demand, including sparkling, non‐sparkling, sugar‐free, sugar‐reduced, and caffeine‐free options (The Coca‐Cola Company 2018, 2019). Its competitor, PepsiCo, claims that *Pepsi Spire* is able to provide a range of up to 1000 beverage options: Consumers first choose a beverage of PepsiCo's portfolio and can then personalize the drink by adding up to three flavor shots, for example, vanilla, lemon, or cherry, to the beverage (PepsiCo n.d.). In both cases, the working principle consists of diluting highly concentrated flavor formulations stored in cartridges with water.

### 4.2 Proposed Process Concept

The literature review has shown that a lack in food choice (food monotony) is an issue in space nutrition, whereas space health risks such as reduction in body mass and a cellular redox regulation might be mitigated by providing suitable bioactive molecules in addition to the astronaut's daily diet. Looking into current trends in the food and beverage industry on Earth, consumers are interested in products that are personalized to their liking (e.g., advanced beverage dispensers) and fortified in order to alleviate personal health problems or reach optimal performance. Combining these findings into a product concept, supplement of bioactive molecules could take place in form of fortified beverage nanoemulsions. Even though emulsions are thermodynamically unstable, their main instability mechanisms for phase separation (i.e., creaming and sedimentation) are driven by gravity. Given sufficient dilution to minimize droplet‐droplet interaction, modeling showed sufficient stability in microgravity and simulated microgravity, and even in gravity, in the case the droplet size small enough for their movement to be dominated by Brownian motion instead of gravity (Schmidt et al. 2025).

Personalization of the beverages in form of nutritional value and flavor to enable personalized health, whereas lessen food monotony could be achieved via an in‐space manufacturing process. Transferring a low‐energy emulsification method into a continuous microfluidic setup could help increase emulsification efficiency via increased fluid control and mass transfer, and the approach of campaign manufacturing enables to use the same setup for all fortified beverage compositions, thereby reducing equipment requirements in number, volume, and mass.

**FIGURE 5:**
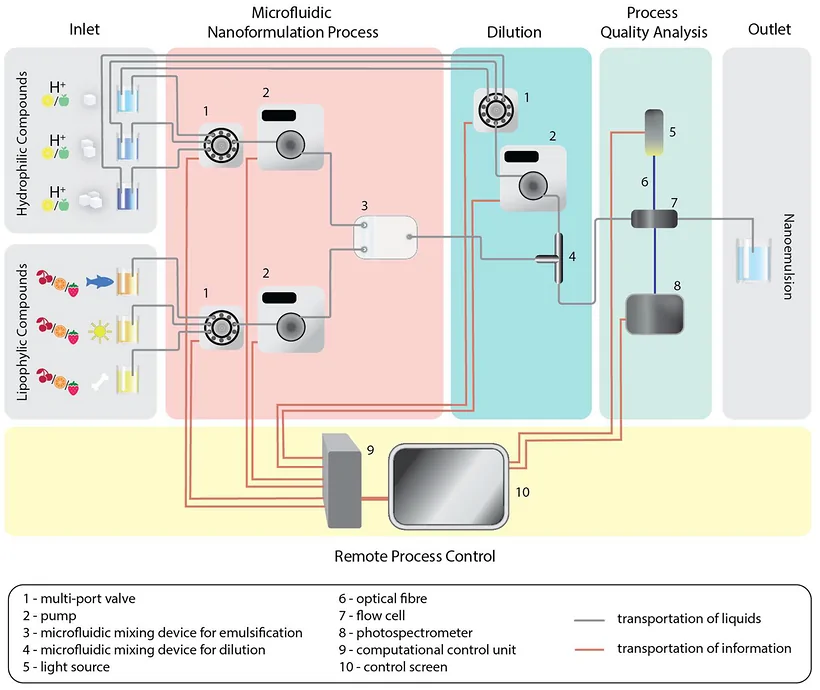
Proposed process concept of an automated in‐space production facility of fortified beverages.

A choice in flavor and nutritional value could be provided in form of a system of “building blocks” (see Figure 5). Hydrophobic compounds, such as the bioactive molecules and odor active compounds (often volatile organic compounds), would be dissolved in different combinations in a mix of carrier oil and surfactant, generating the lipophilic building blocks. Hydrophilic compounds, such as a sweetener and an organic acid (e.g., citric acid and malic acid), which provide taste and give perceived flavor depth to the aroma compounds, would be dissolved in different combination and concentrations (e.g., different sweetness levels) in water, generating the hydrophilic building blocks. Via a multi‐port valve, different building blocks can be assessed and fed into the microfluidic mixer to combine both phases to an emulsion. Depending on the process, it might be required to form emulsions at a higher oil content than 0.1 wt% (typical oil content of beverage emulsions) and dilute it subsequently with the appropriate aqueous phase. This could be the case, for example, if the pumps cannot accurately transport at very small volumetric flow rates, as required for the oil phase. A quality control step is advisable to ensure that quality requirements, for example, the intended droplet size, are maintained. Quality control could be done by measuring the absorbance spectrophotometrically and relating it via calibration curves to the respective droplet size. Other options are in‐line dynamic light scattering instruments for droplet size measurement. The whole process could be controlled via a central computation unit, that is, connected to a control screen. Designing an appropriate software would allow astronauts to choose between bioactive ingredients, flavors, and sweetness/intensity levels on the control screen, with the software automatically determining appropriate volumetric flow rates as well as controlling multi‐port valves, pumps, quality control, and cleaning cycles. Cleaning cycles are especially important to prevent microbial contamination of beverages and equipment failure via fouling of the channels.

In the future, the process could be extended by using personal biomarkers as input into the software to react to nutrient deficiencies in real time and adjust the composition of the beverages accordingly. Furthermore, certain bioactive molecules could be obtained by in‐space processing of plants, bacteria, or animals grown via space farming. Lastly, to embed the process into the recycled water loop in the spacecraft, the process concept needs to be extended with a process step that either dissolves sugar and acid in the aqueous phase or dilutes pre‐brought syrups to appropriate concentrations.

### 4.3 Beverage Emulsions

Regardless of the specific type of beverage, that is, soft drinks, juice‐based fruit drinks, and functional drinks, their formulations can be based upon so‐called beverage emulsions, thus allowing to incorporate hydrophobic compounds like flavor oils, stabilizers, certain nutrients, colorants, and flavors as well as clouding agents that would otherwise separate from the aqueous phase (Tan 2003; Piorkowski and McClements 2014).

**FIGURE 6:**
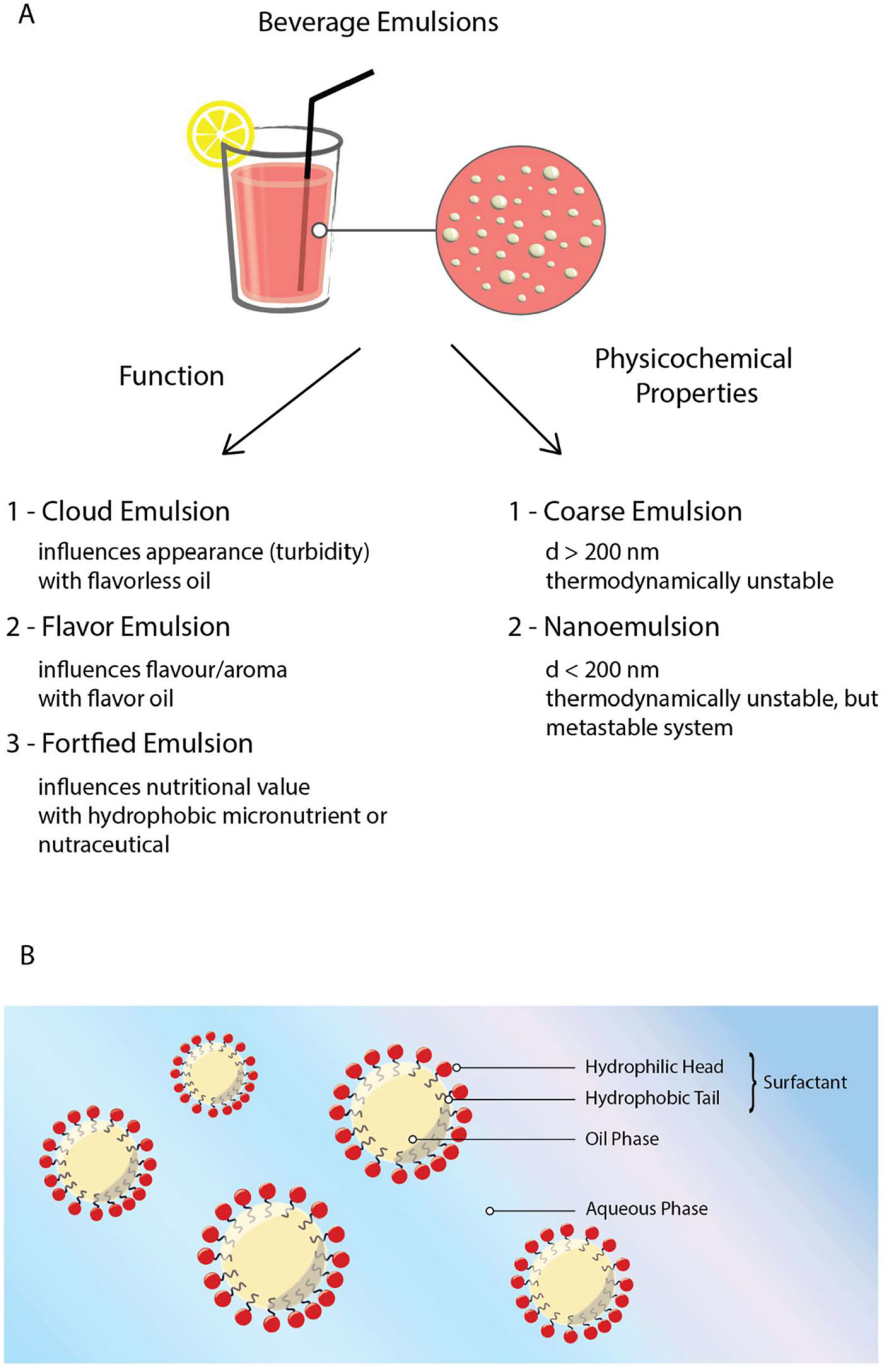
(A) Types of beverage emulsion according to their function or physicochemical properties; and (B) oil droplets dispersed in an aqueous phase with the surfactant forming a layer between oil and water.

A beverage emulsion is a specific type of emulsion, whereas emulsion is a general term used to describe a mixture of two or more liquids that are otherwise immiscible. When viewed on a microscopic or nanoscopic scale, one liquid usually represents the continuous or external phase, thus encasing the droplets of the other liquids that are involved in the emulsion that are referred to as dispersed or internal phase. Therefore, an oil‐in‐water emulsion (o/w emulsion) describes an emulsion consisting of oil as the dispersed phase and water as the continuous phase (see Figure 6B). On contrary, a water‐in‐oil emulsion (w/o emulsion) consists of water as dispersed phase and oil as continuous phase. As mixtures of immiscible fluids relatively quickly unmix without further ado, an emulsifier of some kind is applied to facilitate or enable the formation of droplets and to stabilize the obtained microscopic or nanoscopic structure. Still, beverage emulsions are thermodynamically unstable systems, and emulsion instability mechanisms (gravity‐induced: creaming and sedimentation; induced by droplet–droplet interaction: flocculation, coalescence, and Ostwald ripening) will drive phase separation over time. Products consisting of emulsions—whether they occur naturally or are men‐made—can be found in large numbers in our daily life, with milk being a prominent example in the food section and lotions and creams examples for the cosmetic and pharmaceutical section.

More specifically, beverage emulsions are highly diluted o/w emulsions with the oil phase just constituting to 0.1 wt% of the beverage formulation (Stounbjerg et al. 2017). As for their intended purpose, that is, ingestion, beverage emulsions cannot contain any compound that is identified as being toxic, cancerogenic, or causing an allergic reaction (Vilela et al. 2018). The beverage emulsion compounds can either be a natural food ingredient themselves or certified as “generally recognized as safe” (GRAS) by the FDA or equivalent form local authorities (McClements 2015; Aditya and Ko 2015). The beverage emulsion compounds should be easily available and fulfill labeling requirements (McClements 2015; Patel and Velikov 2011; Tamjidi et al. 2013; Aditya and Ko 2015), while being compatible with the intended application (Aditya and Ko 2015). Likewise, food products need to be equipped with appropriate shelf lives; thus, beverage emulsion must demonstrate stability according to their consumption patterns (Vilela et al. 2018; Tan 2003).

Beverage emulsions can be classified by the intended function they fulfill within the beverage formulation (see Figure 6A). If a beverage emulsion is intended to provide or increase turbidity to the beverage's appearance, then the beverage emulsion is referred to as cloud emulsion. Using a stable, flavorless oil as the dispersed phase, the size of the oil droplets needs to be within the range of the wavelengths of the visible light so that the light is shattered while passing through the emulsion (Piorkowski and McClements 2014). Secondary effects that influence emulsion appearance are droplet concentration (turbidity increases with droplet concentration) and the difference in refracted indices between oil phase and continuous phase (turbidity increases with increasing difference in refractive index) (McClements 2002). Cloud emulsions give a juice‐like appearance to fruit‐flavored beverages, implying a higher concentration of juice to the consumer as is originally present in the beverage (Stounbjerg et al. 2017). In contrast to beverage emulsions, the cloudiness of fruit or vegetable juices is caused by small, dispersed fruit pulp particles (Stounbjerg et al. 2017).

Beverage emulsions can also be applied to enhance flavor of the beverage by encapsulating an flavor agent in the dispersed phase, which is referred to as flavor emulsion (Tan 2003). Popular flavors in the beverage industry are cola, lemonade, and orange‐flavored sodas, all of which require citrus fruits flavoring agents. For citrus fruits, hydrophobic essential oils are obtained from the peel of their fruits (Tan 2003; Piorkowski and McClements 2014). Prior to emulsification, essential oils are typically mixed with a weighting agent to increase the density of the oil phase and to delay creaming (Given 2009).

Finally, fortified emulsions provide a way to encapsulate hydrophobic bioactive compounds, so they can be added as fortified ingredients to a beverage formulation. The handling of bioactives is not simple due to their high chemical instability, and adding bioactives without encapsulation to a food product will not lead to the desired results (Plucinski et al. 2021). When extracted from the food or plant matrix of origin, the concentrated bioactives become increasingly sensible to degradation from environmental conditions, such as pH, oxygen, light, and temperature, and may interact with other ingredient when added to a food, which impacts the stability of the overall food product (da Silva Santos et al. 2019; Plucinski et al. 2021; McClements 2015). Lipophilic bioactives do not mix with aqueous foods but form a film or a sediment instead, depending on their aggregate state at ambient and body temperature (McClements 2015). Adding the bioactives in bulk may even have negative effects on the organoleptic properties (odor, taste) of the food product, and/or impacting its appearance and mouthfeel through aggregates and sediments (Aditya and Ko 2015; McClements 2015). Moreover, simply increasing the concentration of bioactives in the food product will not overcome their shortcomings regarding the bioavailability, which is caused by low bioaccessibility, low absorption, or enzymatic and acidic degradation in the gastrointestinal tract (Norton et al. 2014; Aditya and Ko 2015; McClements et al. 2015). Thus, for the incorporation of bioactive compounds in a food product, a form of encapsulation is essential to prevent their exposure to a potentially destructive environment (Aditya and Ko 2015) but also allows a controlled release at the intended site of action. Although there are other formulation techniques that are interesting for bioactive compounds encapsulation in beverages, such as nanoliposomes, solid–lipid nanoparticles, and nanostructured lipid carriers (Hessel et al. 2023), beverage emulsions are comparatively simple systems of few compounds to apply and due to their widespread use as cloud or flavor emulsion, there is a lot of experience working with this system in the industry, which makes beverage emulsions attractive for bioactive compound encapsulation. The bioactive compound can be added to a mostly chemical inert carrier material, for example, vegetable oil, prior to emulsification. The carrier material's polarity impacts the maximum amount of the bioactives to be encapsulated, whereas its density can make the particles more sensible to gravitational instability mechanisms, and its refractive index impacts the opacity of the beverage (McClements 2015). The emulsion's droplets (size, static charge, etc.) can be specifically designed to control the release of the bioactives at the intended site of action in the gastrointestinal tract, for example, the mouth, stomach, small intestine, or colon (McClements 2015). However, usually the small intestine is the primary site of action for bioactive absorption (Gonçalves et al. 2018). The release at the site of action should be induced by environmental conditions at the site, such as pH, ionic strength, temperature, mechanical forces, or enzyme activities (McClements 2015; McClements et al. 2009).

Apart from function, beverage emulsions can also be classified on the basis of their average particle size. Typically, (beverage) emulsions are divided into conventional emulsion (average droplet diameter >200 nm) and nanoemulsion (average droplet diameter <200 nm) (Tamjidi et al. 2013; Espitia et al. 2019). Compared to conventional emulsions, nanoemulsions are less susceptible to instability mechanisms driven by gravity, leading to a metastable system (Aswathanarayan and Vittal 2019). As the average droplet size is below the wavelength of the visible light, nanoemulsions are clear or slightly turbid (Aswathanarayan and Vittal 2019). Regarding the encapsulation of bioactive molecules, nanoemulsions have shown to provide a higher bioavailability of encapsulated bioactives compared to conventional emulsions, because their higher surface‐area‐to‐volume ratio improves droplet absorption significantly at the preferred site of action (Desai et al. 1996; Tamjidi et al. 2013). Bioavailability of encapsulated bioactives can further be improved by choosing an appropriate composition of the oil phase (Zhang et al. 2016).

### 4.4 Emulsification Process

To obtain a beverage emulsion, the lipophilic phase needs to be dispersed in the hydrophilic phase. However, when mixing two immiscible phases, the interfacial tension between the two phases aims to reduce the interfacial area, and at small quantities of one phase, it assumes the shape of a sphere, as this form provides the lowest interfacial area, comparatively (McClements 2009). The smaller the spherical droplet, the larger is the interfacial area and the interfacial tension. Therefore, to disperse one phase as droplets in another, or to reduce the size of the droplets, approaches need to be applied to overcome or to reduce the interfacial tension. To overcome the interfacial tension, the phases are typically exposed to strong mechanical agitation, whereas the addition of emulsifiers leads to a reduction in interfacial tension but also stabilizes the formed droplets. These processes are called high‐energy emulsification processes and are commonly used in the food industry for different kinds of emulsions (Tan and McClements 2021). Typical devices for high‐energy emulsification processes are high‐pressure valve homogenizers, ultrasonic homogenizer, and microfluidizer (Roohinejad et al. 2018). However, exposure to strong mechanical forces can lead to strong temperature gradients in the mixture, affecting temperature‐sensitive compounds (Roohinejad et al. 2018). Because of the high‐energy requirements, the size of equipment, and the potential degradation of heat‐sensitive compounds, high‐energy emulsification processes are unsuitable for an in‐space production of fortified beverages.

An alternative to high‐energy emulsification processes are low‐energy emulsification processes. Low‐energy emulsification processes do not require a high‐energy input via mechanical forces, as droplet formation is driven by physicochemical characteristics and phase behavior of the surfactant (Roohinejad et al. 2018; Santana et al. 2013). Therefore, compared to high‐energy emulsification processes, low‐energy emulsification processes require less energy input and less specialized equipment, making them easier to implement, while being more efficient at producing emulsions of very small droplet diameter (Yang et al. 2012). However, as these methods rely on the properties of the surfactant, low‐energy emulsification processes require a careful choice in emulsion components. For example, natural‐sourced surfactants, such as proteins or polysaccharides, do not work for low‐energy emulsification (Piorkowski and McClements 2014). Instead, they often require synthetic, small molecule surfactants, potentially in addition of a cosolvent such as ethanol, in the combination of a low‐viscosity oil, which limits choice in emulsion components (Yang et al. 2012; Roohinejad et al. 2018; Santana et al. 2013). Furthermore, typically high surfactant concentrations are required to reliably obtain emulsions (Yang et al. 2012). Still, low‐energy emulsification methods are more suitable than high‐energy emulsification methods for an implementation in space, yet a process needs to be chosen that uses GRAS ingredients at safe concentrations for consumption at the absence of cosolvents, as inflammable solvents represent a hazard, among other issues.

Low‐energy emulsification processes include different variants of the phase inversion method as well as the spontaneous emulsification. Phase inversion methods evoke an inversion of the dispersed phase and the continuous phase by changing a process variable, typically temperature (phase inversion temperature, PIT) or composition (emulsion inversion point, EIP) (Piorkowski and McClements 2014; Perazzo et al. 2015). To obtain an oil‐in‐water emulsion, a water‐in‐oil emulsion would be exposed to changing temperatures or increasing water content until either the properties of the surfactant change to evoke a phase inversion or the oil‐to‐water ratio evokes a catastrophic phase inversion (Piorkowski and McClements 2014). However, applying strong changes in temperature is not suitable for beverage fortification, as the process might destroy temperature‐sensitive compounds. In the case of the emulsion inversion method, factors such as the oil and surfactant type, oil phase composition, surfactant‐to‐oil ratio, initial surfactant location, and, in the case of batch processes, stirring speed influence the droplet size of the final emulsion (Mayer et al. 2013; Ostertag et al. 2012; Aravand and Semsarzadeh 2008). Even though edible nanoemulsions can be reliably obtained via the emulsion inversion method (Ostertag et al. 2012), it is still an emulsification method of at least two steps, that is, forming a water‐in‐oil emulsion, which is then inverted into an oil‐in‐water emulsion. For space application, however, a one‐step emulsification is preferable to minimize required equipment, process time, and risk of potential process failures.

**FIGURE 7:**
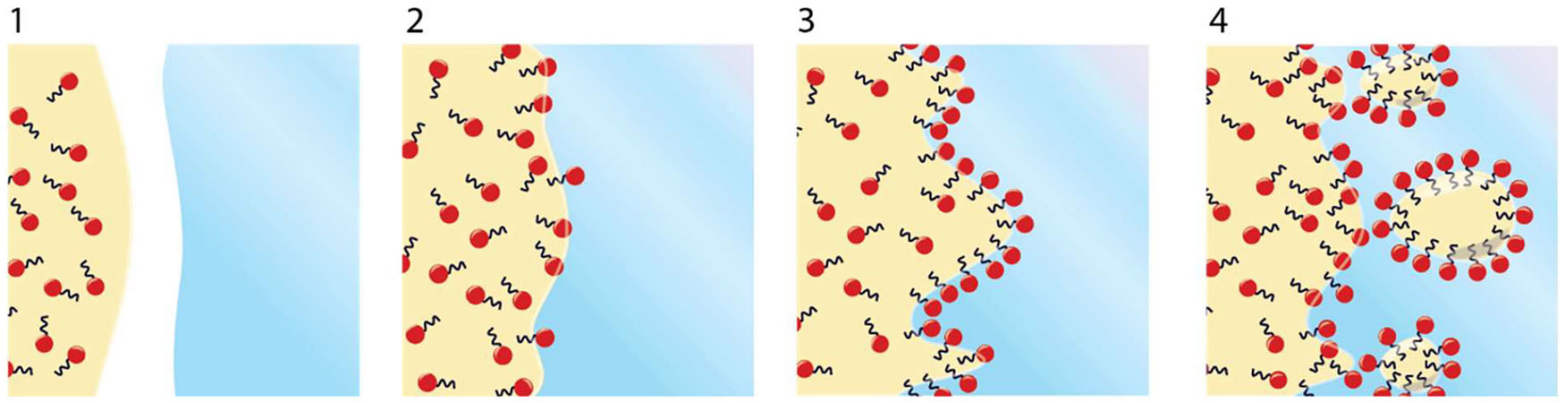
Order of events of the spontaneous emulsification: (1) oil phase and aqueous phase are not in contact, and surfactant is dissolved in the oil phase; (2) oil phase and aqueous phase come into contact with each other; (3) surfactant moves to the interphase; (4) surfactant diffuses to the aqueous phase, carrying along oil droplets. Adapted from Komaiko and McClements (2015).

The other low‐energy emulsification process is the spontaneous emulsification. For this type of emulsification, the position of the surfactant changes over the course of the process (see Figure 7). Prior to emulsification, the surfactant is dissolved in the phase that is intended to be the dispersed phase. Due to the surfactant's properties of being soluble in both phases but having a greater affinity for the continuous phase, it rapidly diffuses into the continuous phase once both phases come into contact, forming spontaneously droplets at the interphase during the mass transfer (Komaiko and McClements 2015; Anton and Vandamme 2009; Pouton and Porter 2008; Horn and Rieger 2001). The surfactants affinity towards the water or oil is described by its hydrophilic–lipophilic balance, and via a suitable choice in surfactant, both oil‐in‐water and water‐in‐oil emulsions can be generated (Gupta et al. 2017). In the case of oil‐in‐water emulsions, additional compounds might be used to aid the droplet formation, for example, a water‐miscible solvent in the oil phase and/or cosolvents and cosurfactant in the aqueous phase (Piorkowski and McClements 2014). Factors such as oil type, oil phase composition, surfactant‐to‐oil ratio, surfactant‐to‐emulsion ratio, initial surfactant location, temperature, and, in the case of batch processes, stirring speed influence the obtained droplet size of the final emulsion (Komaiko and McClements 2015; Saberi et al. 2013). A non‐fortified, edible emulsion made of medium‐chain triglycerides as oil and Polysorbate 80 as nonionic, synthetic surfactant has been reported to reliably form droplets of approx. 100 nm at a surfactant‐to‐oil ratio of 2.0 at 10 wt% oil content (Komaiko and McClements 2015). Therefore, using a simple system of three GRAS compounds is able to form nanoemulsions and might be a suitable solution for in‐space manufacturing. However, surfactant concentrations need to be reduced, as they do not provide any nutritional benefit and are above FDA and European Union regulations (U.S. Food and Drug Administration 2024; European Commission 2024). Furthermore, the oil phase was often added via titration to the aqueous phase (Komaiko and McClements 2015; Saberi et al. 2013), which is not a process setup suitable for space due to the reliance on gravity. Optimizing the surfactant content in the final emulsion and the process setup might enable the application of the spontaneous emulsification in space.

### 4.5 Process Considerations

To enable the in‐space production of fortified beverages via spontaneous emulsification, a suitable process system needs to be identified. Assuming a crew of six people who consume a fortified drinks of 330 mL per day each, the process would need to produce approx. 720 L of beverages in a year. In the case of small annual production quantities (0.01–100,000 t/year), a batch process is typically economically favorable (Grundemann et al. 2009; Holtze and Boehling 2022). As batch reactors have a simple setup, operation, maintenance, and cleaning are relatively easy, making the batch process a suitable option providing a high degree of flexibility for a large range of reactions and processes within a multiproduct plant for chemicals and related products on Earth (Grundemann et al. 2009; Dimian et al. 2014). However, batch processes are unsuited for in‐space manufacturing, as their limited potential for automation requires crew members to conduct and oversee the production, whereas the high cleaning frequency produces large amounts of waste water (Grundemann et al. 2009; Grundemann and Scholl 2014), when both the crew's time and the access to water are already limited. Furthermore, insufficient mixing characteristics and associated low repeatability as well as changing product quality (Grundemann et al. 2009; Grundemann and Scholl 2014) are unlikely to improve emulsification efficiency of the spontaneous emulsification, for example, reduce surfactant concentrations.

The alternative to batch processes is continuous processes, which provide comparatively easy automation and efficient cleaning (Grundemann et al. 2009). Typically, continuous processes are specifically designed for one product at optimum process conditions to achieve large annual production quantities while reducing the amount of byproducts, however, that leads to a lack of flexibility, as the process line is unlikely to be suited for the production of other products (Grundemann et al. 2009; Holtze and Boehling 2022). Different approaches have been proposed to overcome the lack of product flexibility while maintaining the efficiency of continuous‐flow processes. One approach is the modular design of chemical plants, in which modules of fixed size are designed for different unit operations (Kockmann 2015; Roy 2017; Seifert et al. 2012, 2015, 2016). For a new plant, appropriate modules would be chosen and connected, and the process parameter adapted to reach the optimal reaction conditions, thereby significantly lowering planning and construction time and allowing the reusability of modules for different products, thus providing product flexibility. Another approach is campaign manufacturing, that is, the practice of using one continuous plant for the production of a range of similar products, that is, a multiproduct continuous plant (Grundemann and Scholl 2014; Grundemann et al. 2009; Grundemann, Schoenitz, et al. 2012; Sahlodin and Barton 2015). This way, the plant can be adjusted to optimal process conditions for the group of desired products, achieving a high purity and quality of the products. Between the production of the different products, that is, campaigns, a cleaning cycle is initiated to remove any residues of the previous production cycle. The campaign manufacturing approach is promising for the in‐space manufacturing of fortified beverages, as it allows one to produce a range of beverages of different composition using the same setup, thereby reducing equipment needs in the spacecraft. One setup can be used to produce beverages containing different bioactive compounds, flavors, or sweetness levels. Extending campaign manufacturing with a modular setup allows to easily replace and repair malfunctioning modules or to adapt the production potentially to different emulsion‐based or liquid products.

### 4.6 Fluid Manipulation

Fluid manipulation describes the controlled behavior of fluids and is achieved via appropriate equipment. For space exploration, the equipment required for in situ manufacturing should have a small footprint and be lightweight, as additional weight increases the fuel consumption at launch considerably (Hoffman and Kaplan 1997), whereas the room inside the spacecraft is limited (Perchonok et al. 2012). Furthermore, in space, microgravity impacts the behavior of liquids. Natural convection on Earth is often driven by density differences, that is, buoyancy, which is absent in space. On Earth, capillary flow is stopped once the gravitational force acting on the liquids is equal to the forces pulling the liquid up the capillary (given an adequately capillary diameter). In space, however, due to the absence of gravity, the interaction of surface tension and adhesion is able to maintain the spontaneous capillary flow over considerably longer distances at greater capillary diameters, thereby reducing pumping requirements (Nijhuis et al. 2022; Smirnova et al. 2023).

For continuous processes in general, the behavior of fluids is considerably impacted by the diameter of the channeling, as it is one of the parameters describing the flow regime, for example, laminar and turbulent. Continuous reactors with considerably small channeling diameters, that is, 1 µm ≤ *d* ≤ 1 mm, are referred to as microfluidic reactors (Tarn and Pamme 2014; Jähnisch et al. 2004; Cottet and Renaud 2021) and could provide a resource‐efficient solution for small scale in‐space manufacturing. Compared to larger scale continuous processes, in microfluidic processes, fluid behavior is dominated by the viscous forces and the surface tension. The viscous forces result in a laminar flow for most cases, which can be easily predicted via mathematical modeling (Cottet and Renaud 2021). The dominance of surface tension enables capillary flow to some extend in microfluidic devices, and this effect is utilized, for example, in passive, paper‐based microfluidic devices for health diagnostics (Olanrewaju et al. 2018). Due to the small diameters in the microfluidic channels, diffusion times along concentration gradients are very short, improving mass transport (Jähnisch et al. 2004). Additionally, at multiphase flows, the interfacial area is considerably larger, supporting mass transport from one phase into the other (Jähnisch et al. 2004). Thus, the use of microfluidics allows a precise fluid control and efficient mixing. Due to these advantages, microfluidics is currently considered to be a suitable platform technology for in‐space applications, for example, designing compact setups for biological and biochemical experiments in CubeSats satellites (Kuang et al. 2022).

Microfluidic setups can produce uniform droplets reliably via their internal geometry, with droplet sizes being dependant on flow rate ratios and channel dimensions (Shang et al. 2017; Ding et al. 2020). However, without high pressure, that is, high‐pressure microfluidizer (Martz et al. 2012; Villalobos‐Castillejos et al. 2018), nanoemulsion can only be obtained by utilizing additional mass‐transfer effects. For example, applying a solvent variant of the spontaneous emulsification (cosolvent: ethanol, removed after emulsification via dialysis) in a microfluidic mixer resulted in smaller droplets than a comparative ultrasound emulsification process (Fathordoobady et al. 2021). Therefore, using microfluidics as reaction environment for a low‐energy emulsification process has the potential to be a suitable nanoemulsification setup for space.

Microfluidic reactors have been tested for the campaign manufacturing approach. By designing multiproduct plants in microfluidics for products that require small or moderate production capacities, the energy and time efficiency of a continuous process can be further increased, as microfluidic continuous reactors have higher heat and mass transport coefficients compared to macro‐fluidic continuous reactors (Grundemann et al. 2009). For example, the concept of a microfluidic continuous manufacturing plant was tested for the production of writing ink of different colors (Grundemann, Gonschorowski, et al. 2012; Grundemann and Scholl 2014; Grundemann et al. 2009; Grundemann, Schoenitz, et al. 2012). Compared to the associated batch process, a reduction in plant hold‐up and wastewater is achieved, with a possible reduction factor of the later one of up to 1000 (Grundemann et al. 2009). Therefore, microfluidic campaign manufacturing can help save resources such as recycled water during a space mission. Appropriate choice in microfluidic equipment might even prevent the formation of fouling (Grundemann et al. 2012). Otherwise, using a pulsed flow in the cleaning procedure might help to clean layers of fouling (Augustin et al. 2010). Combining the microfluidic campaign manufacturing approach with a modular design, repairs of the setup can be performed by simply replacing a malfunctioning module with a replacement, using, for example, 3D printing, which has already been demonstrated to be feasible as an in‐space manufacturing method (Prater et al. 2019).

### 4.7 Functional Foods and Food Fortification

Many countries have adapted mandatory nutritional information on food packaging, as well as voluntary health information, for example, the “Nutri‐Score” in the European Union (Merz et al. 2024), to inform consumers about the foods’ nutritional properties and their potential impact on health (Ballco and Gracia 2022). Simultaneously, there is an emerging group of consumers with an increased nutritional awareness, which influences their purchasing choices but also pushes new food product development (Guiné et al. 2020; Steinhauser and Hamm 2018). Certain approaches in modern food development go beyond only minimizing or excluding any adverse health effects (i.e., reduced or zero caloric beverages), as food products are demanded to additionally show positive effects on health and well‐being of the consumer (Piorkowski and McClements 2014). For example, consumers prefer to mitigate certain health issues via adjusting the nutritional intake instead of relying solely on drugs (Velikov and Pelan 2008). However, according to the World Food Bank, the consumer's demands even go further than the solely prevention or relief of chronic diseases in the long term: Food products shall provide a favorable instant maximization of physical and mental well‐being in form of, for example, an enhanced mental vigilance, feeling more energetic, or improved gut health (Kotilainen et al. 2006). Thus, so‐called functional foods have to fulfill at least one of the following two health claims: reduce the likelihood to develop a noncommunicable disease and/or promote optimal performance in certain body systems when regularly included at adequate amounts within a healthy diet (Roberfroid 2002; Granato et al. 2020), with any health claim supported by sufficient scientific evidence (Granato et al. 2020). Functional foods represent the link between normal alimentation and medication, as they are consumed in the form of food products but can provide positive effects beyond the conventional nutrition (Velikov and Pelan 2008).

Although the definition of functional foods includes processed foods as well as unprocessed, natural foods, fortified, or enriched foods specifically describe foods which composition is modified to enhance its health promoting properties (World Health Organization and Food and Agricultural Organization of the United Nations 2006). Typically, ingredients added to or increased in the original food composition are probiotics, micronutrients, and nutraceuticals, all of which are also popular as supplements. Probiotics describes the addition of a defined amount (e.g., 1 × 10^9^ colony forming units per serving) of live microorganisms to food items for a health benefit backed by scientific evidence (Hill et al. 2014). The microorganisms are intended to settle and multiply in the gut to enhance the microbial gut environment and support digestive health (Hill et al. 2014; Naseem et al. 2023), although there is evidence that the microbial gut influences many body functions and has even be connected to potentially influence mental health (Madabushi et al. 2023). Microorganisms added are either non‐strain specific, safe species or a defined probiotic strain of a species, and *Bifidobacterium* and *Lactobacillus* are among the microbial species most investigated (Hill et al. 2014; Naseem et al. 2023). Micronutrients are, together with macronutrients, indispensable components of the human nutrition. Although macronutrients (fats, protein, carbohydrate, dietary fibers, and water) need to be consumed in large quantities to provide the body with energy, material for cell growth, and renewal and hydration, micronutrients (essential trace elements and vitamins) are only required in small quantities (Biesalski Hans and Jana 2018; Shenkin 2006). Micronutrients and their metabolites enable a variety of biochemical reactions in the human body by acting as cofactors and coenzymes (Shenkin 2006). Due to their specific role in metabolism, micronutrients cannot replace one another, and any long‐term deficiencies in micronutrients cause severe health issues (Biesalski Hans and Jana 2018; Shenkin 2006). Contrary to micronutrients, nutraceuticals are not essential for survival. The term nutraceutical is a portmanteau of the words “nutrient” and “pharmaceutical” used in food marketing to generally describe bioactive compounds or phytochemicals with evidence of positive effects in physiological function or chronic disease prevention (Nasri et al. 2014; Velikov and Pelan 2008; Palthur et al. 2010). As the term nutraceutical is not properly regulated or defined, the term bioactive compounds is used instead in this literature review. Bioactive compounds are present in natural foods and plants, yet often at low concentrations (Aditya and Ko 2015). Of the low bioactive compounds’ concentration, even less might be absorbed during ingestion, as most of the bioactive compounds show high chemical instability, low solubility in water, low bioavailability, and/or are tightly bound to the food matrix (Weiss et al. 2008; Plucinski et al. 2021), which makes food fortification with bioactive compounds at efficacious levels sensible in order to access their nutritional benefits.

### 4.8 Identifying Micronutrients and Bioactive Compounds Suitable for Beverage Fortification

In the following section, the suitability of specific micronutrients and bioactive compounds for fortification of beverages for future long‐term space travel will be discussed. As hydrophilic compounds can simply be dissolved in the recycled water on the spacecraft (similar to the space beverage powders currently used for tea, coffee, and fruit‐flavored drinks), focus will be laid on hydrophobic compounds for fortification. The beverage fortification with hydrophobic compounds can be implemented using a beverage emulsion as formulation technique. To be suitable for supplementation of space nutrition, any compound needs to be either essential but not available in current space foods or provide a proven health benefit that could not be achieved otherwise.

#### 4.8.1 Micronutrients

Even though micronutrient only account for a small quantity in the human nutrition, they are essential for survival, as they regulate and catalyze many metabolic reactions. Micronutrients consist of nutritional minerals and vitamins, but of these only the vitamins A, D, E, and K are lipophilic (Smith and Zwart 2020). Under the current guidelines for supplementation, however, only vitamin D can be considered for beverage fortification, as it is the only micronutrient whose levels cannot be maintained via regular space food intake. Vitamin D is required for maintaining bone health (Dakkumadugula et al. 2023). It increases the absorption of calcium and phosphorus in the intestine by 30%–40% and 80%, respectively (Holick 2007). Without sufficient vitamin D, bones are undersupplied with calcium and phosphorus, leading to osteoporosis and increased risk of fracture (Holick 2007), thereby amplifying the microgravity‐induced loss of bone. However, as vitamin D deficiency is prevalent among the world's population (approx. in 50% of people during winter), fortification of foods and supplementation with vitamin D is also recommended on Earth (van Schoor et al. 2024). Therefore, fortified beverages containing vitamin D might also be a suitable option to improve the nutritional status on Earth.

When referring to vitamin D, there are two different forms that are nutritionally relevant: Vitamin D2 (ergocalciferol) from certain plant‐sourced foods, and vitamin D3 (cholecalciferol) from either certain animal‐sourced foods or formed by UV exposure in the human skin from the precursor 7‐dehydrocholesterol (Alayed Albarri et al. 2022; van den Heuvel et al. 2024). Both vitamin D forms are enzymatically hydroxylated to serum 25‐hydroxyvitamin D, whose level is typically assessed in the blood to determine the individual vitamin D status (Alayed Albarri et al. 2022; van Schoor et al. 2024). Both are commercially available as supplements (van den Heuvel et al. 2024), yet astronauts are typically given vitamin D3 supplements (NASA 2010), as it has a considerably higher potency compared to vitamin D2 (Armas et al. 2004; van den Heuvel et al. 2024). Even though vitamin D2 is less potent, it might prove as a valuable supplement to maintain adequate serum‐25 dehydroxyvitamin D levels in the future of space exploration, as it can be produced in situ as part of the space farming approach via UV radiation of mushrooms and yeasts (Keegan et al. 2013).

Due to the widespread vitamin D deficiency on Earth, there is effort to fortify a range of foods and beverages with vitamin D by encapsulating it in appropriate nano‐ or microstructure to overcome issues, such as low water solubility, chemical instability, and low bioavailability (Maurya et al. 2023). Encapsulation of vitamin D3 in nanoemulsions via low‐energy emulsification is possible and led to average droplet sizes of 25–70 nm (phase inversion) (Maurya and Aggarwal 2019) and >200 nm (spontaneous emulsification) (Guttoff et al. 2015). Encapsulation in the nanoscale range of vitamin D3 in gum arabic showed a higher bioavailability in rats compared to nonencapsulated vitamin D (Lamsen et al. 2020). Fortification of fruit juices and milk with encapsulated, powdered vitamin D showed that apple juice, orange juice, and whole milk as food matrix provided better bioavailability in a simulated human digestion than skim milk and the control of fortified phosphate buffer (Fu et al. 2024). Regular consumption of milk and orange juice fortified with vitamin D (encapsulation method in both cases not disclosed) led to an increase of 25‐hydroxyvitamin D (Tangpricha et al. 2003) as well as improved bone health (Daly et al. 2006) in study participants. Therefore, it is not only possible to encapsulate vitamin D in a beverage nanoemulsion, but a proper food matrix might increase bioavailability and can improve 25‐hydroxyvitamin D levels in the blood, leading to stronger bones.

#### 4.8.2 Bioactive Compounds

Bioactive compounds are compounds that occur naturally at low concentrations in foods (plants, animals, and microbes) but might provide health benefits (e.g., preventing disease) exceeding their nutritional value when consumed regularly in specified amounts (Sharif and Khalid 2018). For example, melatonin, well‐known to regulate sleep, has recently been connected to delaying tumor growth, among other anti‐inflammatory and antioxidant properties, and is considered for supplementation during cancer treatment (Arnao and Hernandez‐Ruiz 2018; Bhattacharya et al. 2019; Kamfar et al. 2024). Micronutrients may have a bioactive compound function if the amounts required for an effect are above the amounts consumed within a regular nutrition. In relation to space travel, supplementation of bioactive compounds would be beneficial if they could counteract health risks associated to life in space, for example, loss of bone, oxidative stress, and decrease in appetite (see Table 1).

**TABLE 1:** Overview of lipophilic micronutrients and bioactive compounds for potential beverage fortification.

Compound	Supplementation as	Category	Suitability for supplementation	Proposed benefit in space	Against space hazard	Evidence/Current status	Limitations/Remarks
**Vitamin D3**	Micronutrient	Vitamin	Yes	Maintenance of vitamin D status	Lack of natural vitamin D	Nanoemulsions improve bioavailability	Current astronaut supplementation in form of tablets
**Vitamin D2**	Micronutrient	Vitamin	Yes	Maintenance of vitamin D status	Lack of natural vitamin D	Possible future in‐space production form mushrooms	Lower potency than D3; not specifically studied in astronauts
**Vitamin A**	Bioactive compounds	Vitamin	Inconclusive	Antioxidant; anti‐inflammatory	Oxidative stress; space radiation	Associated with increased mortality in some studies	Not specifically studied in astronauts
**Vitamin E**	Bioactive compounds	Vitamin	Inconclusive	Antioxidant; anti‐inflammatory	Oxidative stress; space radiation	Associated with increased mortality in some studies	Not specifically studied in astronauts
**β‐Carotene**	Bioactive compound	Carotenoid	Inconclusive	Antioxidant	Oxidative stress, space radiation, cancer	Supplementation associated with increased mortality in some studies	Not specifically studied in astronauts
**Polyphenols and phytochemicals**	Bioactive compounds	Plant bioactives	No	Antioxidant; anti‐inflammatory	Oxidative stress, space radiation, cancer	Better supplied through fruits and vegetables	Complex mixtures preferred over isolated compounds
**Resveratrol**	Bioactive compounds	Polyphenol	Potential	Antioxidant; anti‐inflammatory	Oxidative stress, space radiation	More research required	Not specifically studied in astronauts
**Curcumin**	Bioactive compounds	Polyphenol	Potential	Antioxidant; anti‐inflammatory	Oxidative stress, neuroinflammation	More research required	Not specifically studied in astronauts
**Melatonin**	Bioactive compound	Hormone	Potential	Sleep regulation, anti‐inflammatory, antioxidant, antitumor effects	Space radiation, cancer risk, irregular circadian rhythm	Emerging evidence	Not specifically studied in astronauts
**Medium‐chain triglycerides**	Bioactive compound	Lipids	Potential	Increased food intake (with fish oil)	Reduced appetite/space anorexia	Improved quality of life and caloric intake in cancer patients when combined with fish oil	Not specifically studied in astronauts
**Omega‐3 polyunsaturated fatty acids**	Bioactive compound	Lipids	High potential	Reduced bone resorption, anti‐inflammatory, antioxidant, appetite stimulation, neuroprotection, cardiovascular health	Bone loss from microgravity	Mixed results for bone protection; promising for several other effects; appetite stimulation: clinical evidence in cancer patients; stable nanoemulsions available	Susceptible to lipid oxidation, especially under radiation

Supplementation with certain bioactive compounds is subject of several countermeasure studies to space‐related health risks, typically performed as a bed rest study on Earth. Bed rest studies are studies during which participants lie for extended periods of time, for example, 30–70 days, in a horizontal or 6° head‐down position, which simulates the redistribution of body fluids in the body as well as the loss of muscles and bones in microgravity (Cromwell et al. 2018; Hargens and Vico 2016; Sundblad et al. 2016). Other space health risks, for example, the increased oxidative stress, cannot be simulated with a bed rest study (Morgan et al. 2012). Still, bed rest studies are currently the most suitable way to test countermeasures to space‐related body changes in the human body on a large scale.

Some bioactive compounds have been identified to potentially enhance bone health (Tang et al. 2022). For example, omega‐3 polyunsaturated fatty acids have been related to a decreased bone resorption in rats and in humans (Griel et al. 2007; Watkins et al. 2003). Omega‐3 polyunsaturated fatty acids are a group of long‐chain fatty acids containing several double bonds (first double bond at omega‐3 position in the main carbon chain), which provides antioxidant properties, for example, α‐linolenic acid, stearidonic acid, eicosapentaenoic acid, docosapentaenoic acid, and docosahexaenoic acid (Shahidi and Ambigaipalan 2018). Omega‐3 polyunsaturated fatty acids cannot be synthesized by the human body but are nutritionally accessible in certain food, especially sea foods (Tur et al. 2012). However, countermeasure studies focusing on omega‐3 fatty acids gave mixed results: Although early bed rest and limited space flight data suggest a decrease in bone loss, recent bed rest studies supplementing omega‐3 polyunsaturated fatty acids together with other bioactive compounds (polyphenols, vitamin E, and selenium) in form of an antioxidant cocktail did not find an effect on bone structure or bone resorption (Austermann et al. 2021, 2023). This antioxidant cocktail did also not help against muscle atrophy (Arc‐Chagnaud et al. 2020). Currently, the best countermeasures against loss of bones and muscles remains resistance exercise in combination with a healthy diet. If viable, an increased intake of fruit and vegetables is suggested to be beneficial to reduce the production of endogenous acid, which, in turn, seems to negatively affect bone health (Frassetto et al. 2018; Zwart et al. 2018).

Antioxidants have also been considered as countermeasure for cosmic radiation and increased oxidative stress. Although shielding of spacecraft is considered the main countermeasure against cosmic radiation, there is no shielding material yet that provides complete protection, resulting in a residual radiation exposure for astronauts (Durante 2014; Hellweg et al. 2020; Montesinos et al. 2021). Therefore, additional countermeasures against cosmic radiation exposure are considered. Even though pharmaceutical options such as amifostine, which is prescribed during cancer treatment, are more efficient for radiation protection than nutritional antioxidants, they might still be preferred to active pharmaceutical ingredients, because nutritional antioxidants have very few, if any, side effects (Zwart et al. 2021; Weiss and Landauer 2003; Hellweg et al. 2020). Nutritional antioxidants are, for example, omega‐3 fatty acids, vitamin E, vitamin C, β‐carotene, selenium, folic acid, and resveratrol, among other (Tang et al. 2022; Montesinos et al. 2021). However, the interactions of nutritional antioxidants on oxidative stress, radiation, and cancer are still little understood to give conclusive recommendations for space‐travel supplementation and food fortification (Hellweg et al. 2020; Zwart et al. 2021). For example, a meta study suggested an increased mortality upon supplementation with β‐carotene, vitamin A, and vitamin E (Bjelakovic et al. 2007). Some studies also focus on food extracts from, for example, green tea or fruits (Montesinos et al. 2021; Hellweg et al. 2020), as these extracts can contain a variety of compounds with antioxidant and bioactive properties, which might be beneficial over supplementing only one compound, as a study feeding a supplement of complex composition to mice extended their lifespan (Lemon et al. 2005). Long‐term space radiation exposure might be particularly hazardous for the brain due to radiation‐induced neuroinflammation, which has been connected to cognitive impairment, memory loss, and changes in behavior, which could considerably impact the success of a long‐term space mission (Peters 2006; Zwart et al. 2021). It is suggested to focus on anti‐inflammatory compounds in the space nutrition as a countermeasure, and many compounds with antioxidant properties can also provide anti‐inflammatory effects, for example, curcumin, omega‐3 fatty acids, resveratrol, folic acid, and vitamins A, C, D, E; yet more research is required (Zwart et al. 2021; Jakubczyk et al. 2020). Phytochemicals, which are a group of bioactive compounds found in plants, especially in fruit and vegetables of bright color, are expected to play an important role in future space nutrition, as they are known for their strong antioxidant and anti‐inflammatory effects (Zwart et al. 2021; Weiss and Landauer 2003). As fruit and vegetables contain a range of phytochemicals, it is recommended to consume fruits and vegetables themselves, or fruit extracts (Guan et al. 2021; Zwart et al. 2021), underlining the importance of space farming.

Furthermore, to the authors’ best knowledge, no studies have so far been performed looking at supplementation of astronauts to specifically stimulate appetite. Supplementing an antioxidant, anti‐inflammatory compounds that simultaneously increases appetite could be quite beneficial for long‐term space travel given the previous issues around adequate food intake. Regarding appetite control, more studies have been performed with a focus on decreasing appetite with bioactive compound supplementation motivated by the global obesity pandemic (Ammendola and Scotto d'Abusco 2022; Nijhawan and Behl 2020; Schiano et al. 2024). However, some research has focused on patients suffering from a declining appetite due to cancer treatment or aging (Ukovic and Porter 2020; Cox et al. 2020). In clinical studies, a supplementation of fish oil, sometimes in combination with medium‐chain triglycerides, improved the quality of life of patients suffering from lung cancer and pancreatic cancer considerably by increasing appetite, which led to increased caloric intake (Sanchez‐Lara et al. 2014; Werner et al. 2017).

Therefore, supplementation of omega‐3 fatty acids might not only provide antioxidant and anti‐inflammatory effects but might also increase appetite during space travel. In aged mice, omega‐3 fatty acids have shown to stimulate the formation of new neurons (Jiang et al. 2009), which could furthermore protect the brain from space‐induced damages. In general, omega‐3 fatty acids are well known to decrease the risk of cardiovascular disease and death (Khan et al. 2021). An analog mission of 45 days, which compared an enhanced diet (>6 servings of fruit and vegetables per day and 2–3 servings of fish, among other) to the standard space diet on the ISS, showed that under the enhanced diet, study participants had reduced cholesterol and cortisol levels, better cognitive skills, and a resilient microbiome, therefore recommending an omega‐3 fatty acids‐rich diet among other for long‐term space missions (Douglas et al. 2022). Findings on whether fish oil supplementation is equivalent to omega‐3 fatty acids from fish consumption are inconclusive and might depend on the respective effect (cardiovascular, appetite) (Werner et al. 2017; Maehre et al. 2015; Zibaeenezhad et al. 2017), yet for space travel it is a good alternative in the absence of fresh fish sources, although there is already early‐stage research on aquaponics for space travel (Kitaya et al. 2023).

Encapsulation of omega‐3 fatty acids in beverage nanoemulsions is possible, as demonstrated in a study using phospholipids as surfactant and a microfluidizer for emulsification (Komaiko et al. 2016). Furthermore, omega‐3 fatty acids are not only suitable for spontaneous emulsification using sunflower oil as carrier, Tween 85 as surfactant, and isopropanol as cosolvent, leading to droplet sizes of 100–200 nm, but also show a greater stability against oxidation and bioavailability compared to the bulk oil (Inapurapu et al. 2020). Omega‐3 fatty acids are susceptible to lipid oxidation, especially under cosmic radiation, which needs to be considered for beverage fortification (Walker et al. 2015; Salvia‐Trujillo et al. 2021). A systematic review on oxidation of encapsulated omega‐3 fatty acids suggests that lipid oxidation is considerably defined by encapsulation efficiency and particle size of nanostructures (Vieira et al. 2022).

In general, the long‐term stability of bioactive compounds in space remains an under‐investigated subject. Limited stability studies are available for both bioactive compounds and micronutrients in a matrix (liquid and dry supplement formulations, food item). Flight opportunities to orbiting space laboratories (both automated and manual) are scarce, whereas it is still difficult to reproduce space radiation on Earth, however, the increased availability of satellite technology might accelerate stability studies. Still, prior to widespread adaptation of bioactive supplementation their stability in bulk and formulated should be studied to ensure the anticipated effects are not diminished by degraded bioactives.

## 5 Conclusions

Reliable food systems for long‐term space missions are still in development. As of now, there is no functionable food supply that could enable space missions lasting several years outside the reach of resupply vehicles. The experience in space missions up to today has shown that providing astronauts with adequate nutrition can be difficult, and specific approaches need to be implemented in order to stimulate food intake. Even though the situation has considerably improved, similar problems could re‐arise when implementing novel space food systems for long‐duration space missions, as they rely on considerably different working principles compared to current systems, that is, renewable food supply via farming and cell culturing and in‐space food manufacturing. Therefore, it is detrimental to consider strategies for increasing food acceptability early in the preparation of long‐term space missions.

Looking at the terrestrial food industry, popular approaches such as personalization and fortification are proposed to be adapted to new space food systems. Personalization could improve food acceptability by providing more choice in foods, thereby minimizing food monotony. Food items can provide benefits beyond their nutritional value by containing either naturally or artificially (fortification) increased concentrations of bioactive molecules, although additional nutritional studies utilizing appropriate space simulations are required to provide evidence of functionality. This way, space‐related health risks could be mitigated, thereby, improving health and well‐being in space. For example, increased consumption of omega‐3 fatty acids can reduce risk in cardiovascular disease, increase bone formation, increase appetite, and reduce the effect of oxidative stress as its function as antioxidant. One way of providing astronauts with supplementation of vitamin D and bioactive compounds is in the form of fortified beverage nanoemulsions. Due to their small droplet size, nanoemulsions provide increased bioavailability of encapsulated compounds. Personalization in particular can be achieved by appropriate automation of in‐space manufacturing, whereas a suitable process for the in‐situ production of space beverages has been identified to be a multiproduct continuous microfluidic process in combination with a low‐energy emulsification such as a solvent‐free version of the spontaneous emulsification. This way, one production line can be used for a variety of beverage recipes (campaign manufacturing) at a small foodprint (microfluidics) using less resources than comparative approaches (spontaneous emulsification: no external, mechanical forces; microfluidics: improved mass transfer, capillary flow reduces pumping needs; campaign manufacturing: reduces wastewater from cleaning cycles).

## Author Contributions

**Svenja Schmidt**: conceptualization, investigation, visualization, writing – original draft, writing – review and editing. **Ian Fisk**: writing – review and editing, funding acquisition, supervision. **Ni Yang**: writing – review and editing, supervision. **Maria Saarela**: writing – review and editing, supervision. **Volker Hessel**: conceptualization, writing – review and editing, funding acquisition, supervision.

## Conflicts of Interest

The authors declare no conflicts of interest.
